# Three-Dimensional Descriptions of π/π, CH/π, and Halogen/π Interactions Formed between Phenyl Substructures

**DOI:** 10.1021/acs.jpcb.6c03632

**Published:** 2026-08-05

**Authors:** Daichi Hayakawa, Hiroaki Gouda

**Affiliations:** Department of Biophysical Chemistry, 13059 Showa Medical University Graduate School of Pharmacy, 1-5-8, Hatanodai, Shinagawa-ku, Tokyo 142-8555, Japan

## Abstract

π/π, CH/π, and halogen/π interactions are key intermolecular interactions in molecular design for drug discovery and materials science. Three-dimensional (3D) characterization of these interactions is essential for rational molecular design, as molecules typically form noncovalent interactions in multiple directions within complex systems. Accordingly, 3D descriptions of π/π, CH/π, and halogen/π interactions provide valuable insights for rational molecular design. In this study, we present experimentally and theoretically validated 3D descriptions of interaction-formable regions for π/π, CH/π, and halogen/π interactions around phenyl and halogenated phenyl substructures, based on quantum mechanical molecular interaction field (MIF­(QM)) calculations and database analyses. O-shaped or C-shaped π/π interaction regions are observed above and below the aromatic rings of benzene and halobenzene molecules. Halogen substitution enhances the stability of π/π interactions by 1.0–2.5 kcal/mol. Spherical CH/π interaction regions are located beyond the π/π interaction regions, which are more spatially extended than those of CH/π interactions. In addition, halogen/π and CH/π interaction regions are distributed along the extension lines of C–X (X = Cl, Br, I) and C–H bonds, respectively. The proposed 3D descriptions of π/π, CH/π, and halogen/π interactions provide a framework for understanding and predicting molecular recognition in protein–ligand systems.

## Graphical abstract

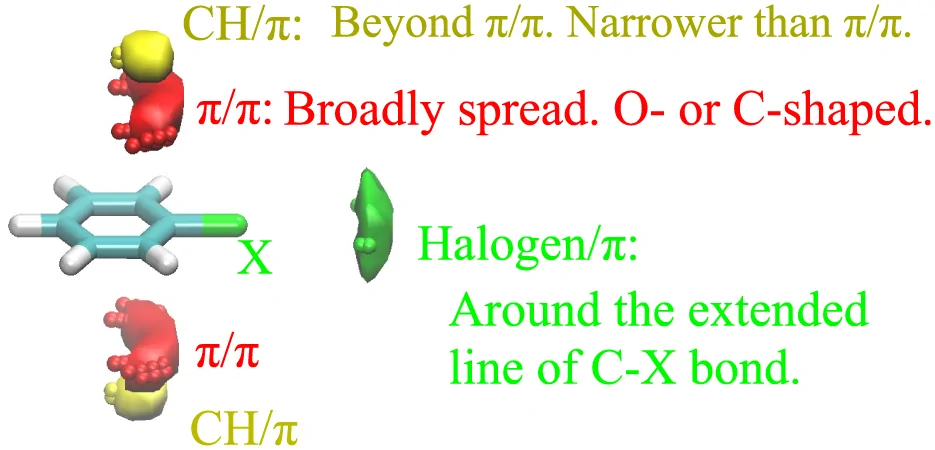

## 1 Introduction

Noncovalent interactions between chemical groups involving π-systems—such as π/π,^1–4^ CH/π,^5–7^ and halogen/π ^8–10^ interactions—play essential roles in stabilizing molecular conformations, crystal packing, and molecular recognition by biopolymers. These interactions are key contributors to intermolecular interactions in molecular design for drug discovery and materials science.^1,3–10^ Such interactions commonly occur between adjacent aromatic groups, and their types depend on the relative configurations of these groups. Aromatic groups can adopt face-to-face or slipped-parallel configurations, forming typical π/π interactions.^1,3,11–13^ Face-to-face configurations correspond to fully parallel alignment between aromatic rings,^12^ whereas slipped parallel configurations involve lateral displacement (Figure 1a).^1,11–13^ These configurations differ only in the relative positions of the aromatic groups. Previous *ab initio* studies have shown that the slipped-parallel benzene (BZ) dimer is energetically more stable than the face-to-face configuration.^11–13^ In this study, both face-to-face and slipped-parallel configurations are collectively referred to as π/π interactions. Interactions between aromatic groups in perpendicular arrangements are often described as T-shaped π/π interactions (Figure 1b). However, this term refers to a geometric configuration rather than a specific type of noncovalent bond.^7^ Noncovalent interactions in T-shaped configurations can be classified into several types, the most common of which is the CH/π interaction.^5–7^ A CH/π interaction is a weak hydrogen bond between a CH group and π-electrons.^5–7^ In such interactions, the CH group of one aromatic ring acts as the donor, while the π-system of the other acts as the acceptor.^7^ In addition, halogen/π interactions may occur in T-shaped configurations when one aromatic group contains a halogen atom.^8–10^ A halogen/π interaction is a type of halogen bond formed between a σ-hole on a halogen atom and an electron-rich π-system.^14–16^ In this case, the π-electrons act as the halogen bond acceptor.^16^ Given the prevalence of halogenated aromatic systems, halogen/π interactions represent an important class of noncovalent interactions alongside π/π and CH/π interactions.^8–10^ The optimal geometrical parameters and interaction energies of π/π, CH/π, and halogen/π interactions have been extensively studied through crystal structure analyses and *ab initio* calculations.^10–13,16–22^ These studies indicate that dispersion interactions are a dominant contributing factor in these systems.^11,16–18^ Most prior investigations have focused on optimized structures,^16,19,20^ or one- and two-dimensional potential energy surfaces obtained by scanning structural parameters.^11–13,16,22^

**Figure 1:**
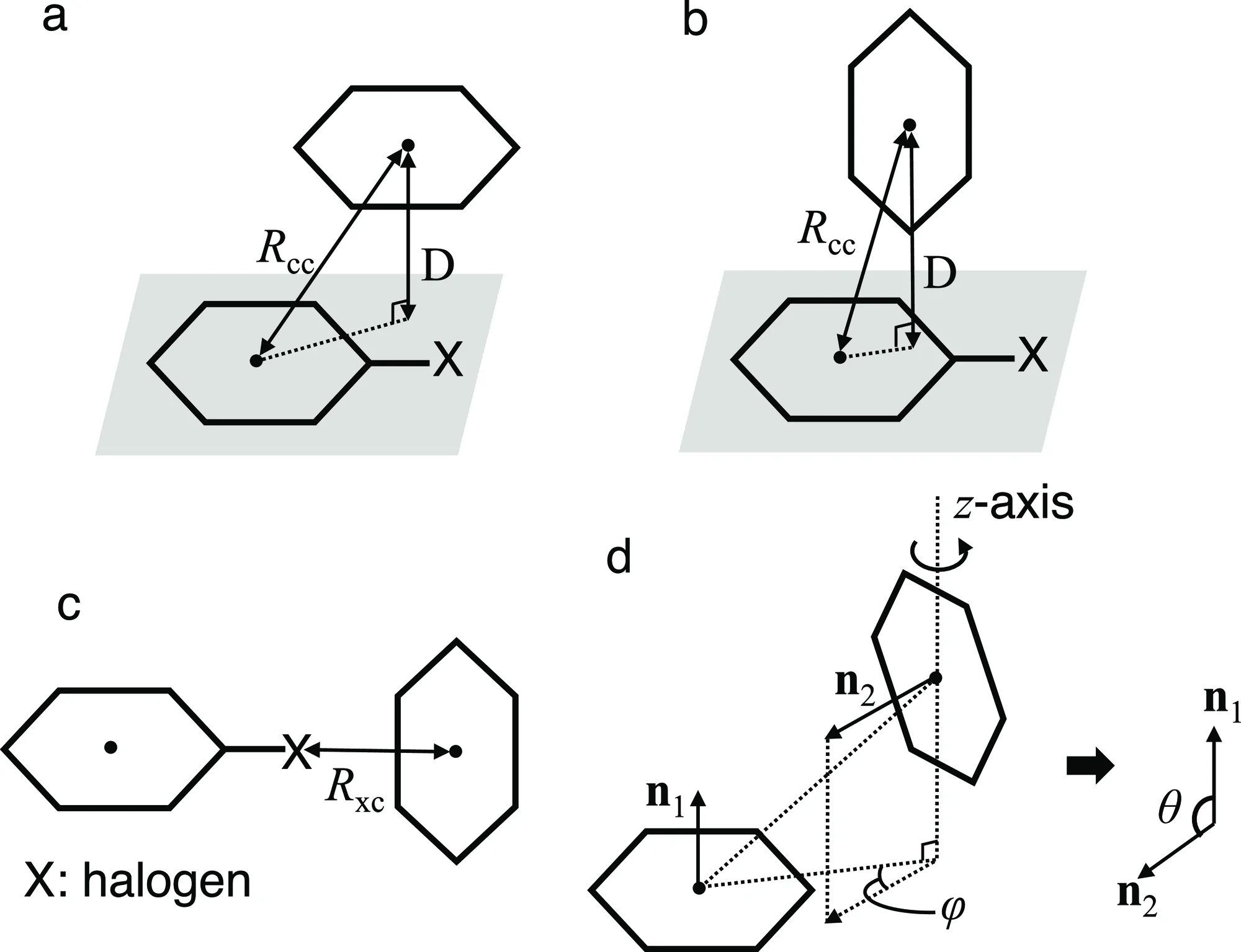
Schematic illustrations of (a) slipped-parallel π/π, (b) CH/π (T-shaped π/π), and (c) halogen/π interactions, along with the corresponding geometric parameters (*R*_cc_, *D*, and *R*_xc_). (d) Definitions of vectors **n**_1_ and **n**_2_ and angles θ and φ. Vectors **n**_1_ and **n**_2_ are normal vectors to the aromatic ring planes. The angle θ is defined as the angle between **n**_1_ and **n**_2_. The angle φ is defined as the angle between (i) the projection of **n**_2_ onto the xy-plane and (ii) the projection of the vector connecting the centers of the aromatic rings onto the xy-plane.

In molecular systems, chemical groups typically form noncovalent interactions in multiple directions, resulting in three-dimensional (3D) interaction networks in crystals, host–guest complexes, and protein–ligand systems. At the local level, these groups often adopt energetically metastable configurations. Therefore, a 3D understanding of noncovalent interactions surrounding chemical groups is critical for rational molecular design. However, studies addressing the full 3D features of π/π, CH/π, and halogen/π interactions remain limited. Recently, we demonstrated that combining 3D potential energy maps derived from quantum mechanical molecular interaction field (MIF­(QM)) calculations with database analyses of crystal structures is an effective approach for investigating the 3D features of intermolecular interactions.^23–25^ In the present study, we elucidate the 3D characteristics of π/π, CH/π, and halogen/π interactions around BZ, chlorobenzene (CBZ), bromobenzene (BBZ), and iodobenzene (IBZ) substructures using MIF­(QM) calculations and database analyses of the Cambridge Structural Database (CSD)^26^ and the Protein Data Bank (PDB).^27^

## 2 Experimental Section

### 2.1 Geometrical Parameters

We define geometrical parameters for π/π, CH/π, and halogen/π interactions (Figure 1a–c). *R*_cc_ is the distance between the centers of aromatic rings (Figure 1a, b). *D* is the perpendicular distance from the center of one aromatic ring to the plane of the other (Figure 1a, b). *R*_xc_ is the distance between the halogen atom of the halogen/π donor and the center of the aromatic ring of the acceptor (Figure 1c). Vectors **n**_1_ and **n**_2_ are normal vectors to the aromatic ring planes. The angle θ is defined as the angle between **n**_1_ and **n**_2_ when their origins are aligned (Figure 1d). The angle φ is defined as the angle between (i) the projection of **n**_2_ onto the xy-plane and (ii) the projection of the vector connecting the ring centers onto the xy-plane (Figure 1d). The parameter φ describes rotation about the *z*-axis.

### 2.2 MIF­(QM) Calculation

The 3D features of π/π, CH/π, and halogen/π interactions were evaluated using MIF­(QM). MIF­(QM) is a 3D potential map that describes interactions around a target molecule and is computed from intermolecular interaction energies between a target and a probe molecule using QM calculations. This approach has been validated in our previous studies^23–25^ and the same procedure was adopted here (Figure 2). Although MIF and related methods are widely used to analyze intermolecular interactions,^28–35^ the present protocol is specifically optimized for π/π, CH/π, and halogen/π interactions. BZ, chlorobenzene (CBZ), bromobenzene (BBZ), and iodobenzene (IBZ) were used as target molecules, and BZ was used as the probe molecule. The calculation consists of five steps: (i) definition of grid points around the target molecule (Figure 2a); (ii) placement of the probe molecule at each grid point (Figure 2b); (iii) orientation of the probe molecule (Figure 2c, d), (iv) QM calculation of the intermolecular interaction energy for the resulting dimer structure, and (v) repetition of steps (ii)–(iv) for all grid points. The interaction energies were normalized relative to the most stable interaction energy. The resulting 3D potential map was visualized as spheres colored according to normalized energy at each grid point (Figure 2e, f). Grid points with normalized energies ≥ 0.9, 0.8, 0.7, 0.6, and 0.5 were colored red, orange, yellow, green, and blue, respectively. Two probe orientations, parallel and perpendicular (T-shaped), were considered (Figure 2c, d). MIF­(QM) maps generated with parallel orientations were used to analyze π/π interactions, whereas those with perpendicular orientations were used for CH/π and halogen/π interactions. In the perpendicular configuration, the probe molecule was oriented such that θ = 90° and φ = 0° (Figure 2d). In this study, parallel and perpendicular (T-shaped) orientations are denoted as “P” and “T,” respectively. For example, the CBZ/BZ­(T) system refers to CBZ as the target and BZ as the probe in a perpendicular orientation. Grid points were distributed over radial distances of 2–7 Å at 0.2 Å intervals, with polar and azimuthal angles ranging from 0° to 180° and 0° to 360°, respectively, in 10° increments, except for the IBZ/BZ­(T) system. For IBZ/BZ­(T), radial distances ranged from 4 to 9 Å at 0.2 Å intervals. This setup yielded a total of 15,964 grid points. Intermolecular interaction energies were calculated using the M06–2X/aug-cc-pVDZ level of theory.^36^ For bromine and iodine, aug-cc-pVDZ-PP pseudopotentials were used, obtained from the Basis Set Exchange.^37^ Basis set superposition error (BSSE) was corrected using the counterpoise (CP) method.^38^ In regions with positive interaction energies (indicative of steric repulsion), normalized energies were set to zero. Similarly, grid points corresponding to failed QM calculations due to severe steric repulsion were assigned zero normalized energy. For stable geometries identified by MIF­(QM), higher-level CCSD­(T) and MP2 calculations were performed to extrapolate CCSD­(T) energies to the complete basis set (CBS) limit using the following equations:

$$\Delta E_{CCSD\left(T\right)/CBS}\approx \Delta E_{MP2/CBS}+\Delta E_{CCSD\left(T\right)/aug-cc-pVTZ(-PP)}-\Delta E_{MP2/aug-cc-pVTZ(-PP)}$$ 1

$$\Delta E_{MP2/CBS}\approx \frac{\Delta E_{MP2/aug-cc-pVTZ(-PP)}X^{3}-\Delta E_{MP2/aug-cc-pVQZ(-PP)}Y^{3}}{X^{3}-Y^{3}}$$ 2

**Figure 2:**
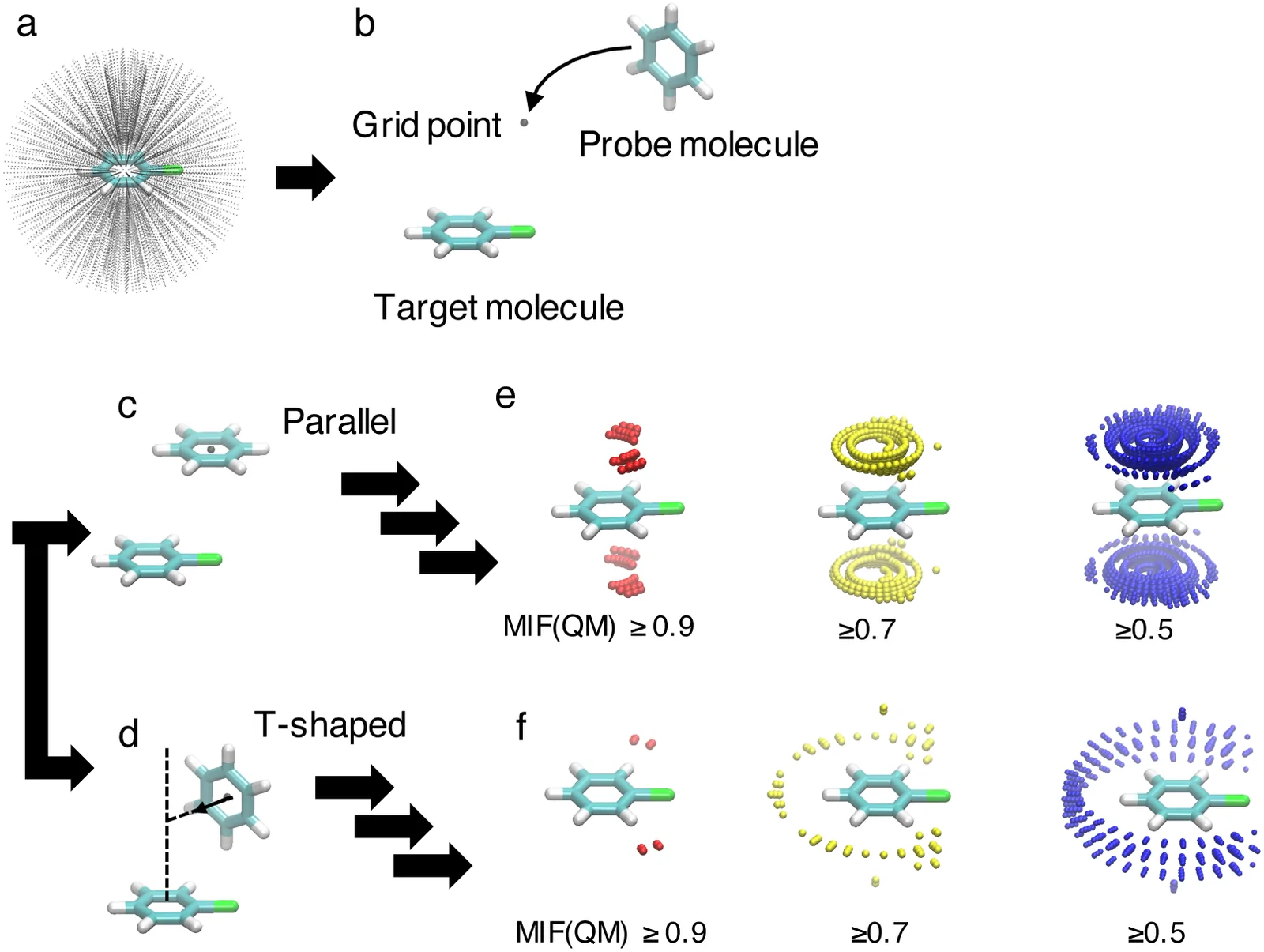
Procedure for MIF­(QM) calculations. (a) Definition of grid points around the target molecule. (b) Placement of a probe molecule. Orientation of the probe molecule: (c) parallel and (d) perpendicular to the target molecule. Examples of MIF­(QM) maps for (e) parallel and (f) T-shaped orientations. Grid points with normalized energies ≥0.9, 0.7, and 0.5 are colored red, yellow, and blue, respectively.

Here, X and Y are cardinal numbers corresponding to aug-cc-pVTZ­(-PP) and aug-cc-pVQZ­(-PP) basic sets (3 and 4, respectively).^32,39^ All QM calculations were performed using the Gaussian16 program.^40^ Probe placement and orientation were controlled using in-house code. Molecular structures were visualized using the Visual Molecular Dynamics (VMD) program.^41^

### 2.3 CSD Analysis

To elucidate the 3D features of π/π, CH/π, and halogen/π interactions based on experimental data, we analyzed crystal structures deposited in the Cambridge Structural Database (CSD).^26,42–48^ Contour density maps (density­(CSD) maps) were generated in this analysis.^42–48^ Density­(CSD) maps are 3D representations of the number density of specific atoms within contact groups surrounding a target (central) atomic group, calculated from interaction data observed in crystal structures in the CSD. These maps represent the statistical probability of locating a contact group at a given spatial position. The overall procedure is illustrated in Figure 3. Contact searches were performed using the CONQUEST program^47^ (Figure 3a). In these searches, atomic groups forming short contacts with the target (central) groups were extracted from the CSD. BZ, CBZ, BBZ, and IBZ substructures were defined as central groups, and BZ substructures were defined as contact groups. Contact searches for π/π, CH/π, and halogen/π interactions were conducted under different conditions, as detailed in the Supporting Information (SI). In this study, two types of CH/π interactions were analyzed separately: those in which the central groups act as acceptors (CH/π-1) and those in which they act as donors (CH/π-2). The centers of contact searches were defined as follows: for π/π interactions, the centers of substructures; for CH/π-1, the centers of aromatic rings; for CH/π-2, the hydrogen atoms of CH groups; and for halogen/π interactions, the halogen atoms (Figure S1). The search radius was set to 7 Å for π/π interactions involving CBZ, BBZ, and IBZ, and to 5 Å for all other analyses. Scatter plots were generated from the resulting contact fragments using the IsoGen program^48^ (Figure 3b). Density­(CSD) maps were then constructed based on the coordinates of these contact group fragments. Although density­(CSD) maps are typically generated using the IsoStar program,^42,48^ in this study, these were generated using in-house code to explicitly account for contact group orientation (Figure 3c). Density­(CSD)­maps for π/π interactions were generated using fragments with parallel orientations (θ ≤ 30° or θ ≥ 150°). Maps for CH/π-1 interactions were generated using fragments with T-shaped orientations (60° ≤ θ ≤ 120°). Maps for CH/π-2 and halogen/π interactions were generated using fragments with T-shaped orientations (60° ≤ θ ≤ 120°) and additional angular constraints on φ (φ ≤ 30° or φ ≥ 150°). The grid interval for density­(CSD) map calculation was set to 0.5 Å. Density values were smoothed by averaging each grid point with its 26 neighboring points. The resulting density values were expressed as percentages relative to the maximum number density (Figure 3c). For comparison with MIF­(QM) maps, density­(CSD) values were calculated based on the coordinates of the centers of contact BZ groups.

**Figure 3:**
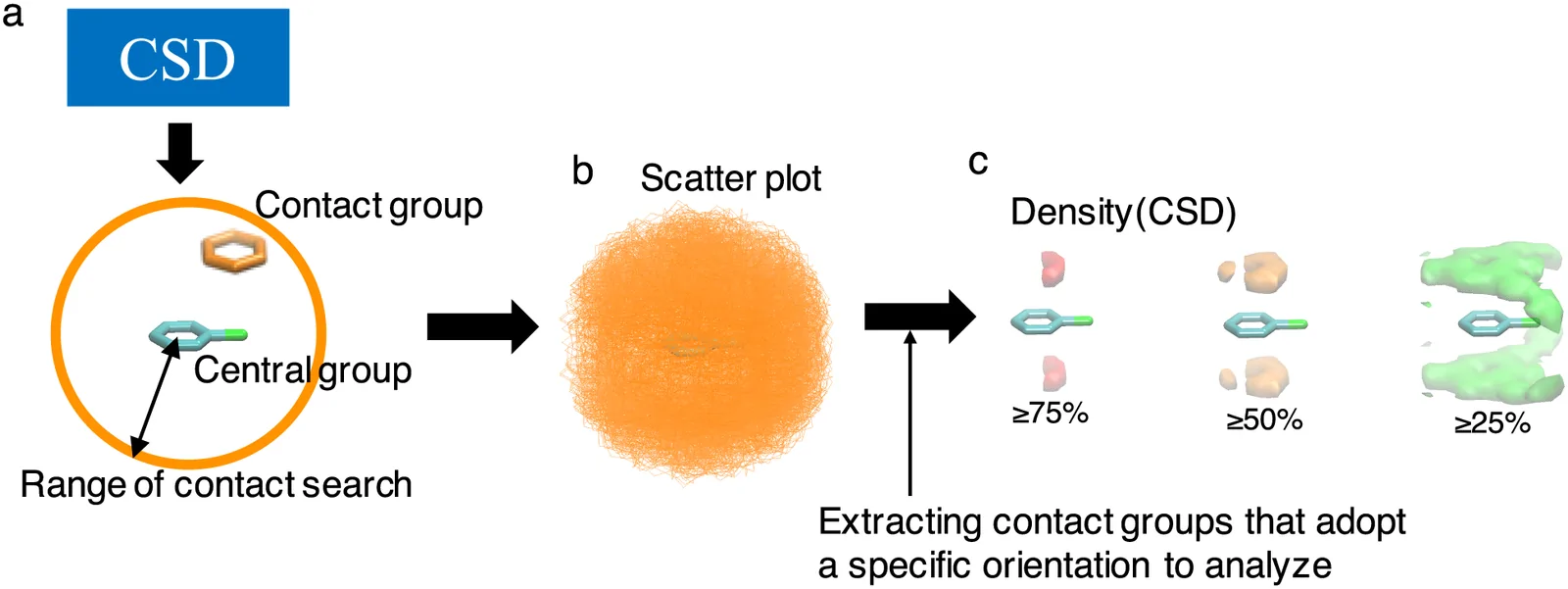
Procedure for generating density­(CSD) maps. (a) Contact search in the CSD. (b) Generation of a scatter plot. (c) Construction of a density­(CSD) map. Examples of density­(CSD) maps with parallel orientation are shown.

### 2.4 PDB Analysis

As in the CSD analysis, π/π, CH/π, and halogen/π interactions around BZ, CBZ, BBZ, and IBZ substructures were analyzed using protein–ligand complex structures from the PDB. Structures containing ligands with BZ, CBZ, BBZ, or IBZ substructures were retrieved from the PDB (https://www.rcsb.org).^27^ For BZ and CBZ systems, only X-ray crystal structures with a resolution of 2.0 Å or better were considered. More than 20,000 structures containing ligands with BZ substructures were initially identified. To reduce the data set size, only structures published after 2023 were included. In addition, only small-molecule ligands containing BZ substructures bound to proteins were retained. For BBZ and IBZ systems, the resolution cutoff was relaxed from 2.0 to 3.0 Å to increase the number of available structures. The coordinates of the complex structures were transformed such that BZ, CBZ, BBZ, or IBZ substructures were aligned to their respective reference structures using the Schrödinger Maestro program (Schrödinger, LLC, New York, NY, 2024). Structures that failed to align were excluded from further analysis. As a result, 1770, 2834, 1053, and 393 X-ray crystal structures containing ligands with BZ, CBZ, BBZ, and IBZ substructures, respectively, were obtained. The aligned structures were analyzed using in-house code to generate scatter plots. The phenyl groups of Phe, the phenol groups of Tyr, and the indole groups of Trp were defined as contact groups. These contact groups were classified as parallel, T-shaped, or other orientations, consistent with the CSD analysis. The search radius was set to 5 Å from the centers of the aromatic rings of BZ, CBZ, BBZ, and IBZ substructures for π/π and CH/π analyses, and to 5 Å from the halogen atoms for halogen/π analyses.

## 3 Results and Discussion

### 3.1 π/π Interactions

Figure 4a–c shows the MIF­(QM), density­(CSD), and superimposed MIF­(QM)/density­(CSD) maps for the π/π interactions of BZ. Figure 4d–f shows the corresponding superimposed MIF­(QM)/density­(CSD) maps for the π/π interactions of the CBZ, BBZ, and IBZ substructures. In the MIF­(QM) maps, regions with normalized energies of 0.9 or higher, corresponding to energetically favorable interaction regions, are shown as red spheres. In the density­(CSD) maps, regions with densities of 75% or higher of the maximum density are shown as red surfaces. The full MIF­(QM) and density­(CSD) maps are provided in Figures S2 and S3 in the SI. In CSD analyses, the thresholds of 75%, 50%, and 25% often yield visually clear figures. The density­(CSD) maps with a 90% threshold are shown in Figure S3 of the SI as examples. The density­(CSD) maps with the 90% threshold show very small regions and are difficult to see, especially for BZ/BZ. We then adopted density­(CSD) maps with a 75% threshold for the following discussion. Figure 4a and c shows that grid points with normalized energies of 0.9 or higher are distributed above and below the BZ plane. These high-energy regions correspond to π/π interaction-formable regions. Because the grid points represent the centers of the probe molecules, the O-shaped distribution indicates that slipped-parallel configurations are more stable than the face-to-face parallel configuration. The distance between the BZ ring plane and the plane defined by the most stable π/π interaction-formable points (*D*) is 3.46 Å (Figure 4c). The distance between the center of the target BZ and the grid point with the highest normalized energy (*R*_cc_) is 4.0 Å. The normalized energy range from 0.9 to 1.0 corresponds to interaction energies of −2.66 to −2.39 kcal/mol, which agrees with CCSD­(T)/CBS values reported in previous studies (−2.46 kcal/mol^11^ and −2.78 kcal/mol^12^). Figure 4c also shows that the π/π interaction-formable regions in the MIF­(QM) map are consistent with the interaction regions observed in the density­(CSD) map. This agreement indicates that the theoretically calculated MIF­(QM) maps are consistent with the experimental observations. In addition, Figures S4 and S5 show density­(CSD) maps for the phenyl/phenyl π/π system, in which the BZ substructure with five hydrogen atoms was defined as both the central and contact group. The density­(CSD) map shows a hole at the center of the phenyl substructure, although the O-shaped distribution is interrupted at the carbon atom lacking a hydrogen atom. This result also supports the preference for the slipped-parallel configuration. For halogenated BZs (CBZ, BBZ, and IBZ), π/π interaction-formable regions with normalized energies of 0.9 or higher are distributed 3.3 Å above and below the aromatic planes and exhibit C-shaped or broken C-shaped distributions (D = 3.3 Å, *R*_cc_ = 3.8 Å; Figure 4d–f). The π/π interaction regions observed in the MIF­(QM) maps are consistent with those in the density­(CSD) maps. The interaction energies in these regions are −3.96 to −3.56 kcal/mol for CBZ, −4.11 to −3.70 kcal/mol for BBZ, and −4.27 to −3.84 kcal/mol for IBZ. Thus, π/π interactions between halogenated BZs and BZ are stronger than those between two BZ molecules by 1.0–2.5 kcal/mol, and the corresponding interaction distances are shorter than those in the BZ/BZ system. Figure 4 further shows that the stability of π/π interactions increases with increasing halogen atomic number. The interaction energies at the stable points were recalculated using approximate CCSD (T)/CBS calculations, and the results are listed in Table 1. The interaction energies for the optimal π/π geometries are −2.42, −3.44, −4.35, and −4.92 kcal/mol for BZ/BZ, CBZ/BZ, BBZ/BZ, and IBZ/BZ, respectively. The interaction energies calculated at the M06–2X/aug-cc-pVDZ­(−PP) level with BSSE correction correlate with those obtained by CCSD­(T)/CBS calculations. Given this consistency, together with the good agreement between the MIF­(QM) and density­(CSD) maps, M06–2X/aug-cc-pVDZ­(−PP) calculations with BSSE correction are valid for qualitative assessment of π/π interaction-formable regions. However, because relatively large errors were observed for the CBZ and IBZ systems, CCSD­(T)/CBS calculations are preferable for quantitative comparison of interaction energies. Accordingly, in the following analyses, interaction energies calculated by CCSD­(T)/CBS are used for quantitative discussion.

**Figure 4:**
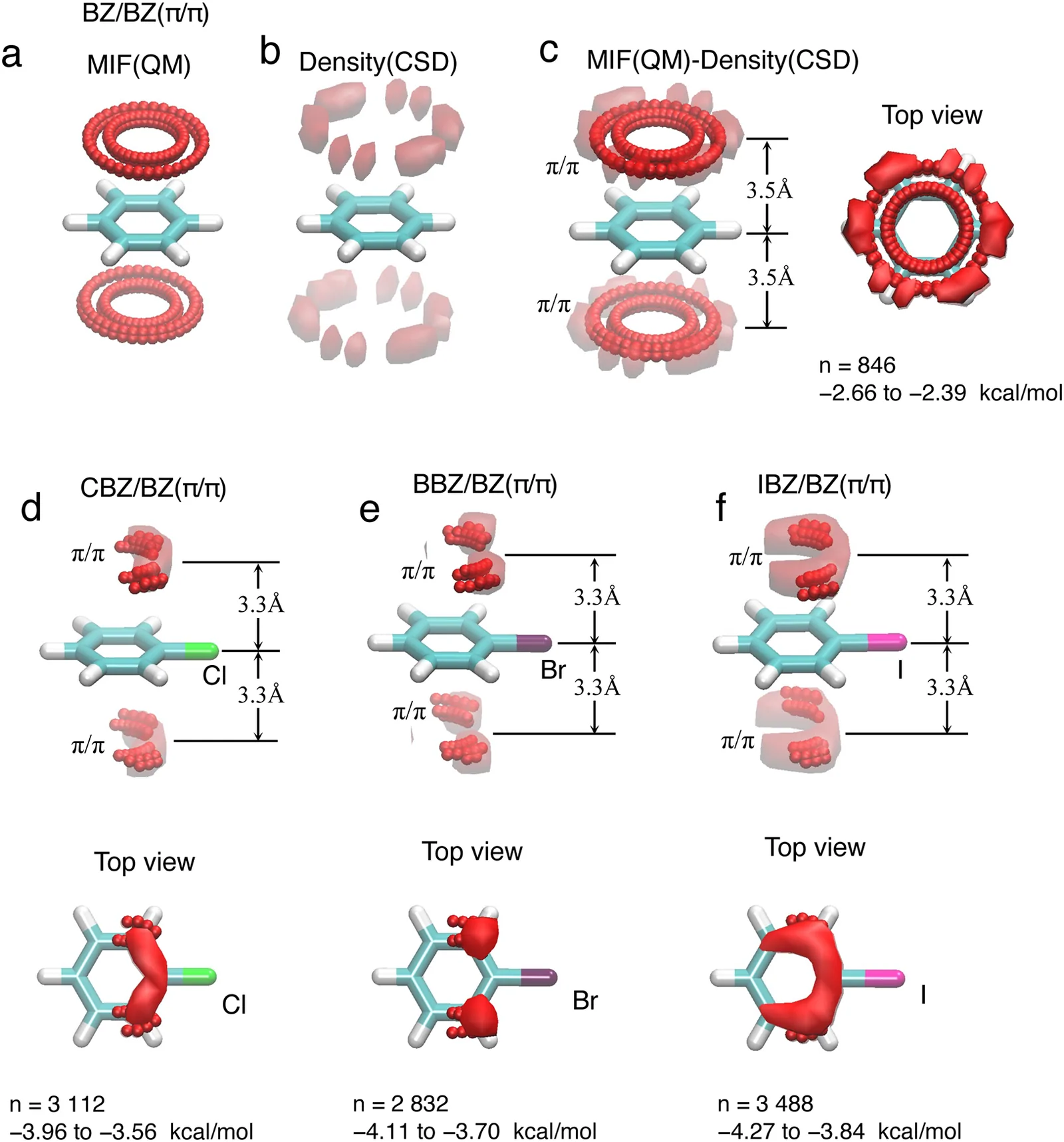
MIF­(QM) (a), density­(CSD) (b), and superimposed MIF­(QM) and density­(CSD)­(c) maps of π/π interactions for BZ. Superimposed MIF­(QM) and density­(CSD) maps of π/π interactions for (d) CBZ, (e) BBZ, and (f) IBZ substructures. In these analyses, probe or contact groups with parallel orientations were used. Grid points with normalized energies ≥ 0.9 are shown as red spheres, and regions with densities ≥ 75% of the maximum density are shown as red surfaces. The contact search range was 5 Å for BZ and 7 Å for CBZ, BBZ, and IBZ. The corresponding non-normalized interaction energies (M06–2X/aug-cc-pVDZ­(−PP) with BSSE correction) for normalized energies of 0.9–1.0 are indicated in the figure. n denotes the number of fragments used to generate the density­(CSD) maps.

**Table 1:** A List of Intermolecular Interaction Energies in the Stable Geometries for π/π, CH/π, and Halogen/π Interactions Obtained by the MIF­(QM) Calculations^a^

Interaction	System	CCSD(T)/CBS	M06–2X/aDZ(CP)^b^	AE^c^	M06–2X/aDZ(nonCP)^d^	AE^e^	RHF/CBS	ΔE_disp_ ^f^
π/π	BZ/BZ	–2.42	–2.66	0.24	–3.61	1.19	3.60	–6.02
	CBZ/BZ	–3.44	–3.96	0.52	–5.19	1.75	5.21	–8.65
	BBZ/BZ	–4.35	–4.11	0.24	–5.35	1.00	5.37	–9.72
	IBZ/BZ	–4.92	–4.27	0.65	–5.53	0.61	5.69	–10.61
CH/π-1	BZ/BZ	–2.42	–2.25	0.17	–3.14	0.72	1.05	–3.47
	CBZ/BZ	–2.57	–2.20	0.37	–3.15	0.58	1.19	–3.76
	BBZ/BZ	–3.03	–2.26	0.77	–3.24	0.21	1.20	–4.24
	IBZ/BZ	–3.43	–2.31	1.12	–3.30	0.13	1.23	–4.67
CH/π-2	CBZ-ortho/BZ	–3.15	–3.06	0.09	–3.97	0.82	1.42	–4.57
	CBZ-meta/BZ	–2.70	–2.63	0.07	–3.70	1.00	1.93	–4.63
	CBZ-para/BZ	–2.74	–2.52	0.22	–3.43	0.69	0.73	–3.46
	BBZ-ortho/BZ	–3.87	–3.14	0.73	–4.07	0.20	1.89	–5.76
	BBZ-meta/BZ	–2.89	–2.65	0.24	–3.71	0.82	1.92	–4.81
	BBZ-para/BZ	–2.74	–2.54	0.20	–3.62	0.88	2.02	–4.77
	IBZ-ortho/BZ	–4.23	–3.37	0.86	–4.44	0.21	2.49	–6.72
	IBZ-meta/BZ	–3.07	–2.66	0.41	–3.73	0.66	1.91	–4.98
	IBZ-para/BZ	–2.84	–2.55	0.29	–3.64	0.80	2.02	–4.86
X/π	CBZ/BZ	–1.58	–1.45	0.13	–1.88	0.30	2.33	–3.91
	BBZ/BZ	–2.74	–2.13	0.61	–2.63	0.11	2.51	–5.25
	IBZ/BZ	–4.22	–2.90	1.32	–3.42	0.80	1.94	–6.15

a The unit is kcal/mol.

b M06–2X/aug-cc-pVDZ­(−PP) with BSSE correction by counterpoise method.

c The absolute error of the interaction energy evaluated by the M06–2X/aug-cc-pVDZ­(−PP) with BSSE correction. The mean absolute error is 0.46.

d M06–2X/aug-cc-pVDZ­(−PP) without BSSE correction.

e The absolute error of the interaction energy evaluated by M06–2X/aug-cc-pVDZ­(−PP) without BSSE correction. The mean absolute error is 0.67.

f The dispersion energy evaluated by the difference between the interaction energy obtained by the CCSD­(T)/CBS and RHF/CBS calculations.

We calculated the interaction energy at the restricted Hartree–Fock (RHF)/CBS level (ΔE (RHF/CBS)) for the stable geometries identified by MIF­(QM) calculations (Table 1). Moreover, the dispersion energies are evaluated as the difference between the energies obtained by CCSD­(T)/CBS and RHF/CBS calculations (Table 1). The interaction energy obtained by RHF consists of electrostatic and polarization-induced attractive energy, and exchange-repulsion energy. ΔE­(RHF/CBS) values of the π/π interactions for BZ/BZ, CBZ/BZ, BBZ/BZ, and IBZ/BZ systems are all positive. It suggests that electrostatic and polarization energy do not cancel out the exchange-repulsion energy. On the other hand, the dispersion energies are highly negative, and the stability increases with increasing halogen atomic number. Furthermore, the dispersion energies correlate well with the total interaction energies as obtained with CCSD­(T)/CBS. It suggests that the π/π interaction energy is stabilized by increased dispersion energy due to halogenation, and that the interaction region becomes C-shaped due to attraction from the halogen atom.

A previous study reported a long-range distribution in the BZ/BZ system where the displacement of the two BZ substructures is from 4.5 to 5.5 Å.^49^ However, that study was based on one-dimensional analysis by scanning geometrical parameters. To verify the present 3D description, we generated a density­(CSD) map within 7Å from the center of the target BZ in the BZ/BZ system. The result is shown in Figure S6. Figure S6 confirms the presence of a more distant distribution at R_cc_ ≈ 5.6 Å, where the displacement of the two BZ substructures is 5.0 Å. The MIF­(QM) map for the BZ/BZ­(P) system suggests that interactions in this region have energies of approximately −1.4 kcal/mol, which is weaker than the interaction energy at the most stable points shown in Figure 4c. This finding suggests that these regions are not major hotspots for noncovalent intermolecular interactions. These distant distributions may arise from second-order effects between molecules imposed by the crystal structure. In contrast, a comparison of the MIF­(QM) and density­(CSD) maps clearly confirms that the O-shaped distribution in Figure 4c originates from π/π interactions. This result highlights the importance of comparing theoretical calculations, such as MIF­(QM), with experimental CSD data. MIF­(QM) and density­(CSD) maps are therefore complementary. In addition, these long-range distributions were not observed for the CBZ, BBZ, and IBZ systems (Figure 4d–f).

### 3.2 CH/π-1 Interactions (Central Groups as Acceptors)

The full density­(CSD) maps for CH/π-1 interactions are shown in Figure S7 in the SI. The full MIF­(QM) maps of BZ/BZ­(T), CBZ/BZ­(T), BBZ/BZ­(T), and IBZ/BZ­(T) are shown in Figure S8. These original MIF­(QM) maps include both CH/π and halogen/π interaction-formable regions. Therefore, we divided these MIF­(QM) maps by interaction type using a simple procedure described in the SI (Figure S9). For simplicity, the divided maps are used in the following discussion.

Figure 5 shows superimposed MIF­(QM) and density­(CSD) maps of CH/π-1 interactions for BZ, CBZ, BBZ, and IBZ substructures. CH/π interaction-formable regions are located 5.0 Å above and below the centers of the aromatic rings in all target molecules (*R*_cc_ = 5.0 Å). The MIF­(QM) maps are in good agreement with the corresponding density­(CSD) maps. These interaction regions are spherical and narrower than the π/π interaction regions. The interaction energies are −2.3 to −2.0 kcal/mol at the M06–2X/aug-cc-pVDZ­(−PP) level with BSSE correction. CCSD­(T)/CBS calculations show that the strength of CH/π interactions increases with increasing halogen atomic number (Table 1). The M06–2X/aug-cc-pVDZ­(−PP) calculations with BSSE correction underestimate the interaction energies for BBZ and IBZ. The agreement can be improved when BSSE correction is not applied.

**Figure 5:**
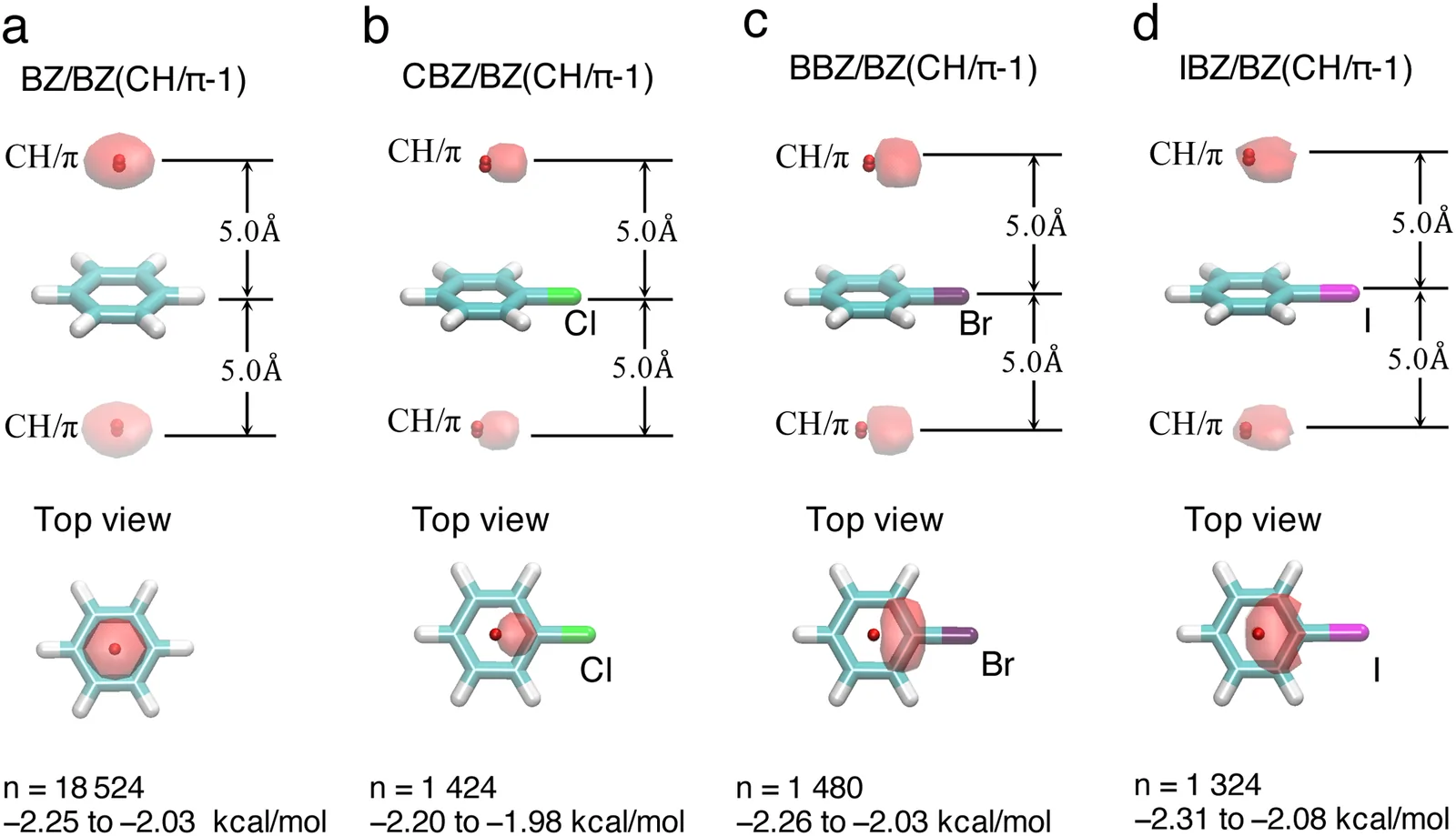
Superimposed MIF­(QM) and density­(CSD) maps of CH/π-1 interactions for (a) BZ, (b) CBZ, (c) BBZ, and (d) IBZ substructures, using probe or contact groups in T-shaped orientations. Grid points with normalized energies ≥ 0.9 are shown as red spheres, and regions with densities ≥ 75% of the maximum density are shown as red surfaces. The corresponding non-normalized interaction energies (M06–2X/aug-cc-pVDZ­(−PP) with BSSE correction) for normalized energies of 0.9–1.0 are indicated. n denotes the number of fragments used to generate the density­(CSD) maps.

Although the MIF­(QM) and density­(CSD) maps of the CH/π interactions show little variation with halogen, the minimal change is also a crucial insight. Furthermore, the consistency between MIF­(QM) and density­(CSD) in CH/π-1 interactions is meaningful for the discussion of scatter plots­(PDB) in the following section. ΔE­(RHF/CBS) values of the CH/π-1 interactions for BZ/BZ, CBZ/BZ, BBZ/BZ, and IBZ/BZ systems are all positive (Table 1). On the other hand, the dispersion energies are highly negative, and stability increases with increasing halogen atomic number (Table 1). The dispersion energy values correlate well with the total interaction energies. It suggests that the CH/π-1 interaction energy is stabilized by increased dispersion energy due to halogenation. The tendency is similar to that of π/π interactions, although the magnitude is lower.

### 3.3 CH/π-2 Interactions (CH Groups of Central Groups Act as Donors)

Figure 6 shows superimposed MIF­(QM) and density­(CSD) maps of CH/π-2 interactions for CBZ, BBZ, and IBZ substructures. Because halobenzene molecules contain five CH groups that can form CH/π-2 interactions, Figure 6 presents MIF­(QM) and density­(CSD) maps for the CH groups at the ortho, meta, and para positions. The full density­(CSD) maps for CH/π-2 interactions are shown in Figure S10–S12. For CBZ, the MIF­(QM) maps of the CH groups at the ortho, meta, and para positions show CH/π-2 interaction-formable regions around each of these CH groups, respectively (Figures 6a, d, g). The grid points with normalized energies of 0.9 or higher are in good agreement with the interaction regions observed in the density­(CSD) maps. The stability order of CH/π-2 interactions around CBZ is ortho-CH > meta-CH ≈ para-CH (Table 1). For the *ortho* CH group of BBZ, the CH/π-2 interaction-formable region with densities of 75% or higher of the maximum density is divided into two clusters (Figure 6b). However, the grid points with normalized energies of 0.9 or higher are included within the region with densities of 50% or higher of the maximum density. The density­(CSD) map for the *meta* CH group of BBZ suggests that CH/π-2 interactions at the ortho position are more stable than those at the meta position (Figure 6e). These results are consistent with the interaction energy order obtained from the MIF­(QM) calculations: ortho-CH > meta-CH > para-CH (Table 1). For the para CH group of BBZ, the grid points with normalized energies of 0.9 or higher are also in good agreement with the interaction regions in the density­(CSD) map. The MIF­(QM) and density­(CSD) maps for the *meta* CH group of IBZ show a trend similar to that observed for BBZ (Figure 6f). The MIF­(QM) maps for the ortho and para CH groups of IBZ are also consistent with the corresponding density­(CSD) maps (Figure 6c, i).

**Figure 6:**
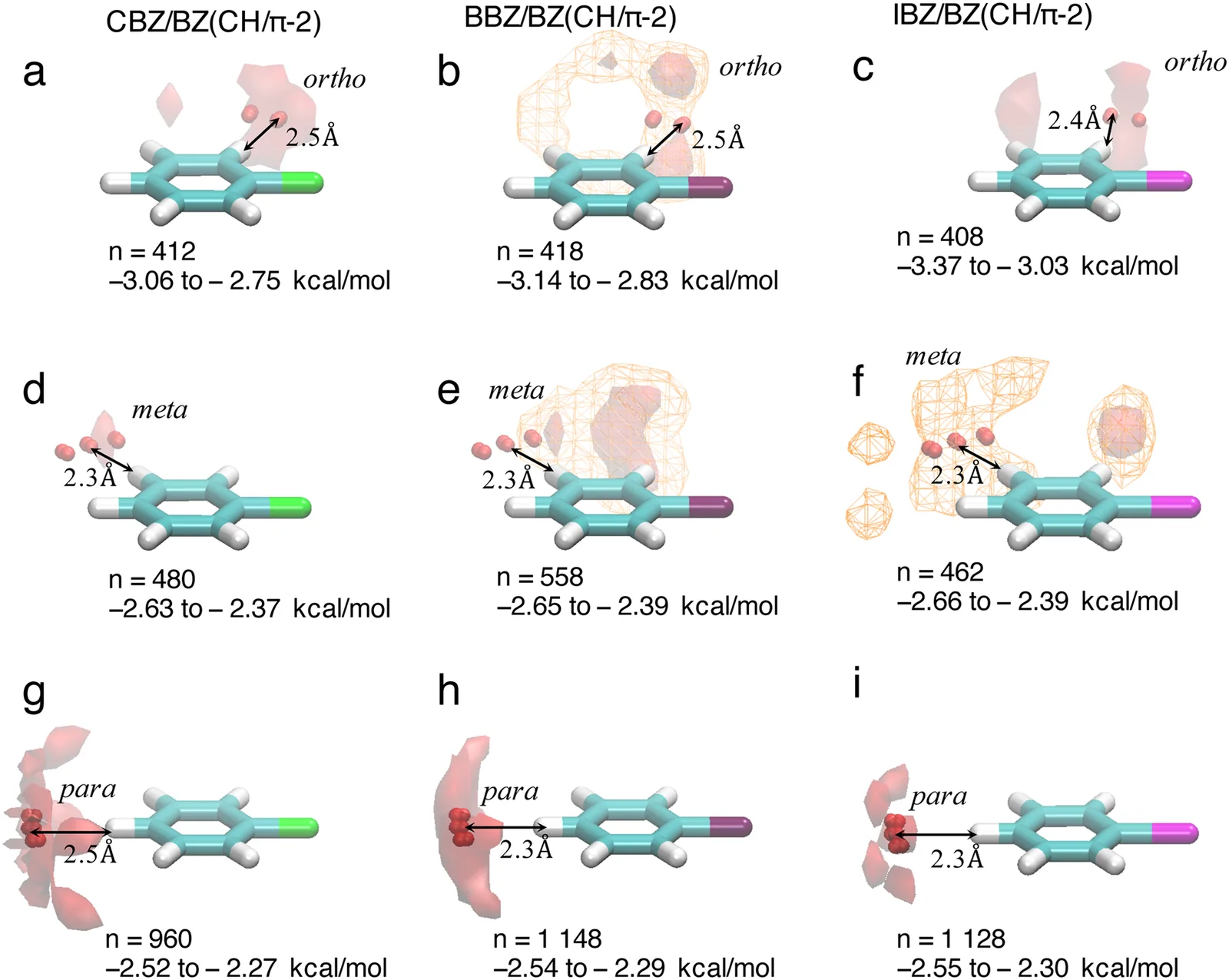
Superimposed MIF­(QM) and density­(CSD) maps of CH/π-2 interactions around ortho-CH groups of (a) CBZ, (b) BBZ, and (c) IBZ, meta-CH groups of (d) CBZ, (e) BBZ, and (f) IBZ, and para-CH groups of (g) CBZ, (h) BBZ, and (i) IBZ. Grid points with normalized energies ≥ 0.9 are shown as red spheres. Regions with densities ≥ 75% of the maximum density are shown as red surfaces, and regions with densities ≥ 50% are shown as orange meshes in (b), (e), and (f). The corresponding non-normalized interaction energies (M06–2X/aug-cc-pVDZ­(−PP) with BSSE correction) for normalized energies of 0.9–1.0 are indicated. n denotes the number of fragments used to generate the density­(CSD) maps.

The MIF­(QM) and CCSD­(T) calculations show that the stability order of CH/π-2 interactions around CBZ, BBZ, and IBZ is ortho-CH > meta-CH ≥ para-CH (Figure 6 and Table 1). The stabilities of CH/π-1 interactions, in which the halobenzene acts as the acceptor, are comparable to those of CH/π-2 interactions at the meta and para positions, in which the CH groups of the halobenzene act as donors (Table 1). Furthermore, the 3D features of CH/π-2 interactions are similar among CBZ, BBZ, and IBZ (Figure 6), whereas their stabilities increase with increasing halogen atomic number (Table 1).

For the CBZ/BZ system, the dispersion energies do not correlate with the total interaction energy (Table 1). The stability of the total CH/π-2 interaction energy seems to depend on the balance between RHF and dispersion terms because the van der Waals radius of the chloride atom is small. On the other hand, in the case of BBZ/BZ and IBZ/BZ, the total CH/π-2 interaction energies at ortho-, meta-, and para-positions correlate well with the dispersion terms (Table 1). The total CH/π-2 interaction energy is the most stable at the ortho position, where the dispersion force from the bromine or iodine is the strongest.

### 3.4 Halogen/π Interactions

Figure 7 shows superimposed MIF­(QM) and density­(CSD) maps of halogen/π interactions for CBZ, BBZ, and IBZ substructures. The full density­(CSD) maps for halogen/π interactions are shown in Figure S13. Halogen/π interaction-formable regions are located along the extension lines of the C–X bonds (X = Cl, Br, I). The 3D features are similar among CBZ, BBZ, and IBZ. The MIF­(QM) maps are in good agreement with the corresponding density­(CSD) maps. The distances between the halogen atom and the most stable halogen/π interaction point (*R*_xc_) are 3.38, 3.43, and 3.63 Å for the CBZ/BZ, BBZ/BZ, and IBZ/BZ systems, respectively. The interaction energies obtained from CCSD­(T)/CBS calculations for the most stable grid points are −1.58, −2.74, and −4.22 kcal/mol for the CBZ/BZ, BBZ/BZ, and IBZ/BZ systems, respectively (Table 1). These results indicate that the interaction energies of halogen/π interactions increase with increasing halogen atomic number. The calculated interaction energies are also in good agreement with values reported in previous CCSD­(T)/CBS studies.^50,51^ M06–2X/aug-cc-pVDZ­(−PP) calculations with BSSE correction underestimate the interaction energies of halogen/π interactions, especially for the BBZ/BZ and IBZ/BZ systems (Table 1). The agreement can be improved when BSSE correction is not applied. Our MIF­(QM) and density­(CSD) maps confirm that the 3D features of halogen/π interactions are similar among CBZ, BBZ, and IBZ, whereas the interaction strengths and interaction distances (*R*_xc_) depend on the halogen species. In addition, the total X/π interaction energies of CBZ/BZ, BBZ/BZ, and IBZ/BZ systems correlate well with their dispersion terms (Table 1).

**Figure 7:**
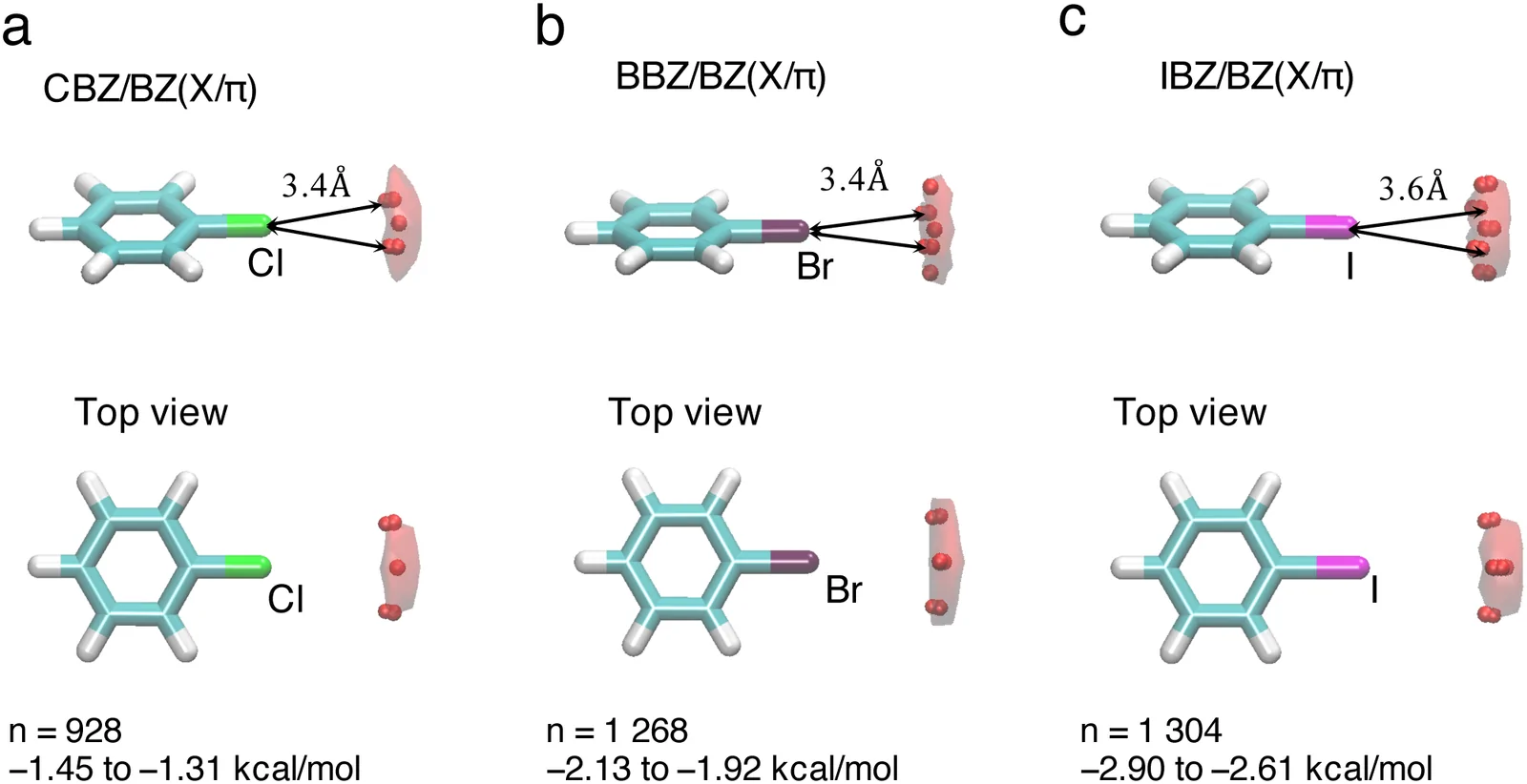
Superimposed MIF­(QM) and density­(CSD) maps of halogen/π interactions for (a) CBZ, (b) BBZ, and (c) IBZ substructures. Grid points with normalized energies ≥ 0.9 are shown as red spheres, and regions with densities ≥ 75% of the maximum density are shown as red surfaces. The corresponding non-normalized interaction energies (M06–2X/aug-cc-pVDZ­(−PP) with BSSE correction) for normalized energies of 0.9–1.0 are indicated. n denotes the number of fragments used to generate the density­(CSD) maps.

### 3.5 Comparisons Among π/π, CH/π, and Halogen/π Interactions and PDB Analyses

Figure 8a compares the π/π and CH/π-1 interaction-formable regions around BZ. The π/π interaction-formable regions are located in planes approximately 3.5 Å from the BZ plane, whereas the CH/π interaction-formable regions are located beyond the π/π interaction regions, at approximately 5.0 Å from the center of BZ. These 3D descriptions are consistent with the *D* and *R*_cc_ values reported in previous studies based on one-dimensional energy surfaces obtained by scanning geometrical parameters.^11,12^ Figure 8b–d compares π/π, CH/π-1, and halogen/π interaction-formable regions around halobenzene molecules. The features of the π/π and CH/π-1 interaction-formable regions are similar among CBZ, BBZ, and IBZ. The π/π interaction-formable regions are located in planes approximately 3.3 Å from the aromatic ring planes of the halobenzenes. The CH/π-1 interaction-formable regions are located beyond the π/π interaction regions, at approximately 5.0 Å from the center of the aromatic ring. The CH/π interaction-formable regions are spherical and narrower than the π/π interaction regions. In addition, the halogen/π interaction-formable regions are located along the extension lines of the C–X bonds (X = Cl, Br, I). CH/π-2 interactions are also formed around the CH groups of halobenzene substructures, although these are not shown in Figure 8b–d. The interaction energy order is π/π > CH/π > halogen/π for the CBZ/BZ system. For the BBZ/BZ and IBZ/BZ systems, the order is π/π > CH/π ≈ halogen/π.

**Figure 8:**
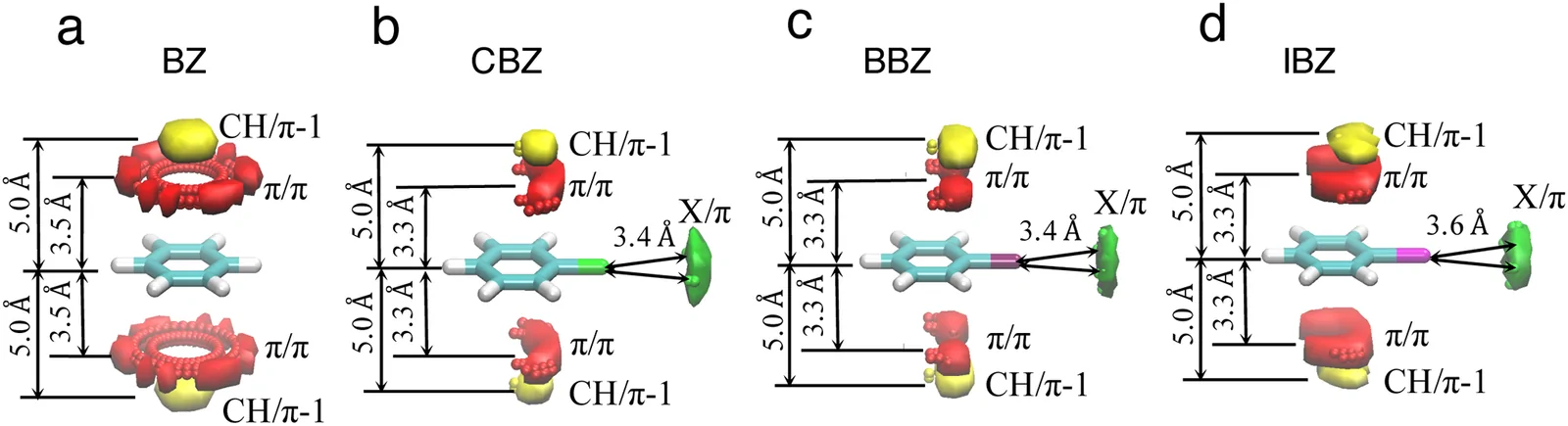
(a) Comparison of π/π and CH/π-1 interaction-formable regions around BZ. Comparison of π/π, CH/π-1, and halogen/π interaction-formable regions around (b) CBZ, (c) BBZ, and (d) IBZ. Grid points with normalized energies ≥ 0.9 are shown as spheres, and regions with density­(CSD) ≥ 75% of the maximum density are shown as surfaces. π/π, CH/π-1, and halogen/π interaction-formable regions are colored red, yellow, and green, respectively.

Figure 9 shows scatter plots (PDB) of BZ contact groups derived from Phe around the BZ, CBZ, BBZ, and IBZ substructures in protein–ligand systems. The centers of the contact searches were set at the centers of the central groups. In addition, scatter plots (PDB) of phenol and indole contact groups derived from Tyr and Trp around the BZ, CBZ, BBZ, and IBZ substructures are shown in Figures S14 and S15.

**Figure 9:**
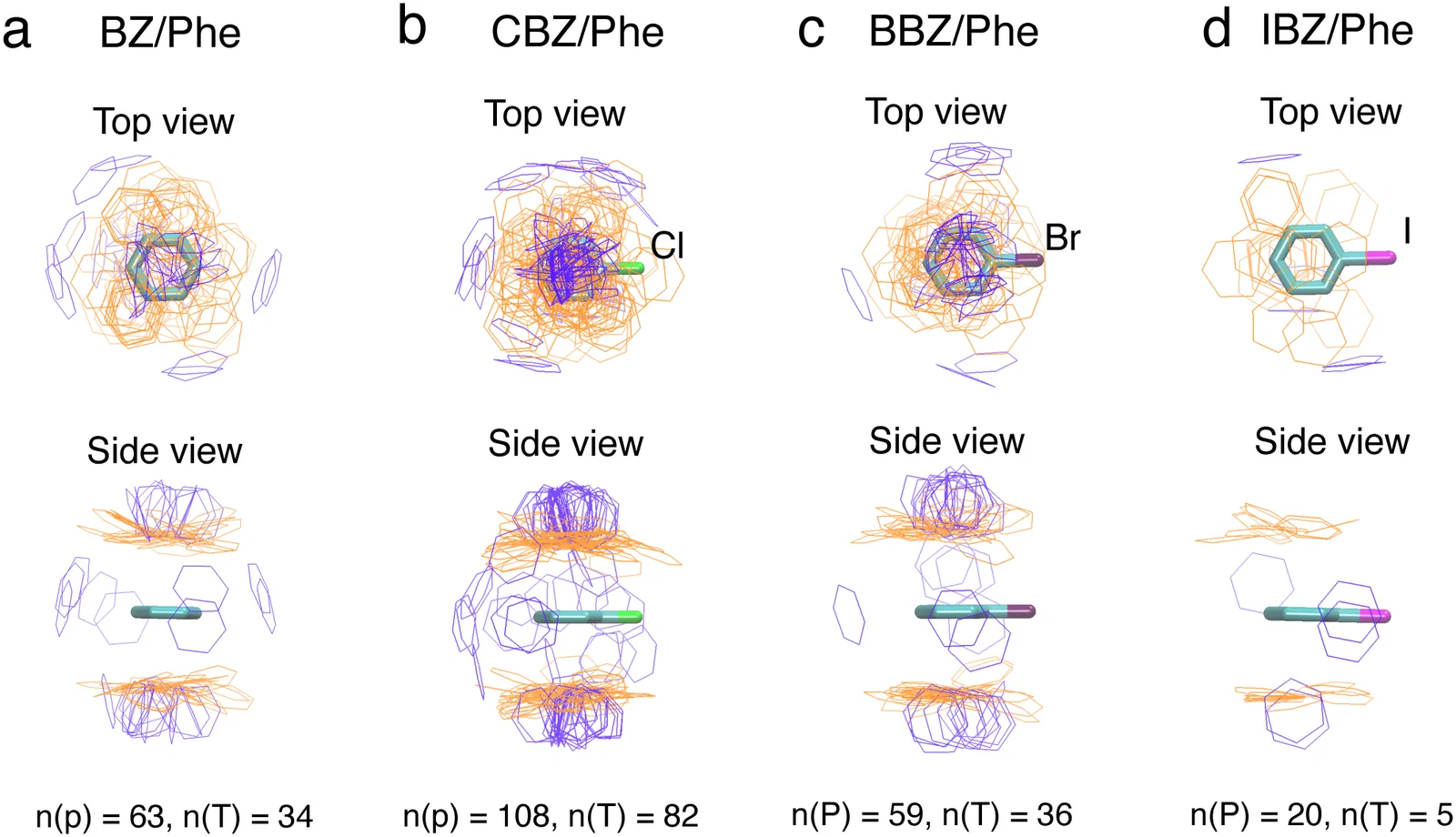
Scatter plots (PDB) of BZ contact groups derived from Phe for (a) BZ, (b) CBZ, (c) BBZ, and (d) IBZ substructures. Contact groups with parallel and T-shaped orientations are shown in orange and purple, respectively. The centers of contact searches were set at the centers of the central groups. n­(P) and n­(T) denote the numbers of fragments with parallel and T-shaped orientations, respectively.

All contact BZ groups with parallel orientations, and most contact BZ groups with T-shaped orientations, are observed above and below the aromatic rings of the central groups (Figure 9). Contact BZ groups with parallel and T-shaped orientations form π/π and CH/π-1 interactions with the central groups, respectively. As an exception, only five BZ groups with T-shaped orientations were found around the aromatic ring of IBZ because of the limited sample size. As in the density­(CSD) maps, contact BZ groups with parallel orientations are located close to the aromatic ring of the central groups, whereas contact BZ groups with T-shaped orientations are located farther from the ring than the parallel ones. In addition, BZ groups with parallel orientations are distributed over a wider region than those with T-shaped orientations. These results show that the 3D features of π/π and CH/π-1 interactions in protein–ligand systems are consistent with those derived from the MIF­(QM) and density­(CSD) maps. Figure 10a–c shows scatter plots (PDB) of BZ, phenol, and indole contact groups derived from Phe, Tyr, and Trp residues around the chlorine atom of the CBZ substructure. The scatter plots (PDB) for BBZ and IBZ substructures are shown in Figures S16 and S17. Contact groups distributed along the extension lines of the C–X bonds are clearly observed (Figure 10). These contact groups form halogen/π interactions with the central groups. In particular, halogen/π interactions between CBZ substructures and phenol groups are clearly observed (Figure 10b). Contact groups not expected to form halogen/π interactions are also present in the scatter plots (Figure 10, and Figures S16 and S17). This observation suggests that halogen/π interactions compete with other interactions, such as CH/X^52^ or CH/π-2 interactions. This trend is reasonable because the stabilities of halogen/π, CH/X, and CH/π-2 interactions are comparable.

**Figure 10:**
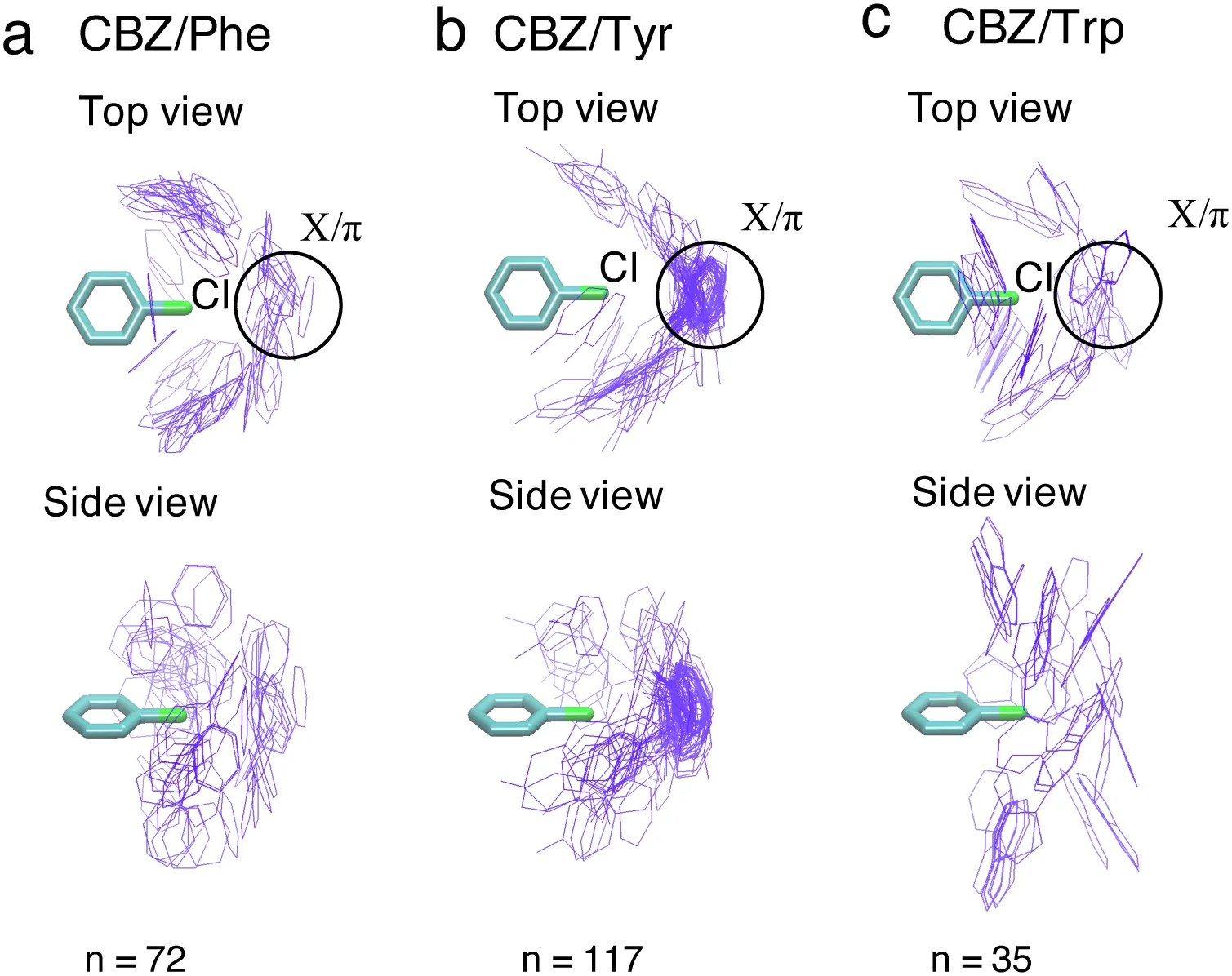
Scatter plots (PDB) of (a) phenyl groups of Phe, (b) phenol groups of Tyr, and (c) indole groups of Trp around the chlorine atom of CBZ. The centers of contact searches were set at the chlorine atoms. Contact groups with T-shaped orientations are shown in purple. n denotes the number of fragments used to generate the scatter plot.

## 4 Conclusion

We present 3D descriptions of π/π, CH/π, and halogen/π interactions around BZ, CBZ, BBZ, and IBZ based on MIF­(QM) calculations and CSD and PDB analyses. O-shaped π/π interaction-formable regions are located approximately 3.5 Å above and below the BZ ring. In CBZ, BBZ, and IBZ, C-shaped or partially broken C-shaped π/π interaction-formable regions are observed approximately 3.3 Å above and below the aromatic rings. The interaction energies of π/π interactions with favorable configurations are −2.42, −3.44, −4.35, and −4.92 kcal/mol for the BZ/BZ, CBZ/BZ, BBZ/BZ, and IBZ/BZ systems, respectively. Halogen substitution increases the stability of π/π interactions by 1.0–2.5 kcal/mol, and the stability further increases with increasing halogen atomic number. CH/π-1 interaction-formable regions are located approximately 5.0 Å above and below the aromatic rings, which act as acceptors, beyond the π/π interaction regions. MIF­(QM) and density­(CSD) maps show that CH/π-1 interaction regions are narrower than π/π interaction regions. CH/π-2 interactions, in which CH groups of halobenzene molecules act as donors, are located along the extension lines of C–H bonds. The stability order is ortho-CH > meta-CH ≥ para-CH. The 3D features of CH/π interactions are similar among BZ, CBZ, BBZ, and IBZ, whereas their stabilities increase with increasing halogen atomic number. Halogen/π interaction-formable regions are located along the extension lines of C–X bonds (X = Cl, Br, I). The 3D features of halogen/π interactions are similar among CBZ, BBZ, and IBZ, whereas the interaction strengths and interaction distances (*R*_xc_) increase with halogen atomic number. Across the investigated systems, MIF­(QM) maps are consistent with density­(CSD) maps. Scatter plots derived from PDB analyses are also consistent with the MIF­(QM) and CSD results, indicating that the proposed 3D descriptions accurately capture the features of these interactions.

In protein–ligand systems, ligand functional groups often adopt energetically metastable geometries at the local level. Binding geometries are determined by multiple factors, including the shape of the binding pocket, the intrinsic conformation of the ligand, and the balance of intermolecular interactions. For example, a CBZ group in a ligand may form a π/π interaction with a phenyl group of Phe when the binding geometry is favorable. However, when such an arrangement is not feasible, CH/π or halogen/π interactions may form instead, as their optimal interaction distances (*R*_cc_) are longer than those of π/π interactions. The 3D descriptions of π/π, CH/π, and halogen/π interactions presented here provide a useful framework for predicting ligand–protein interactions and have broad potential applications in understanding molecular recognition and in drug design.

## Supplementary Material

























## Data Availability

The list of the obtained MIF­(QM) energies, the adopted molecular coordinates for QM calculations, and the list of the PDB IDs used in the analyses are available in the Supporting Information.

## Notes

Details of CSD analyses, division of MIF­(QM) maps, and additional figures (PDF)
List of numbers of hit structures and fragments in CSD and PDB analyses (XLSX)
(XLSX)
List of interaction energies at stable configurations (XLSX)
List of interaction energies obtained from MIF­(QM) calculations (XLSX, XLSX, XLSX, XLSX)
(XLSX)
Coordinates of molecular models used in the MIF­(QM) calculations (ZIP)
List of PDB IDs of crystal structures used in the analyses (XLSX)
(ZIP)
(ZIP)
(ZIP)
(ZIP)

## ABBREVIATIONS

BZ: benzene
CBZ: chlorobenzene
BBZ: bromobenzene
IBZ: iodobenzene
π/π: pi–pi interaction
CH/π: carbon–hydrogen to pi interaction
CH/π-1: carbon–hydrogen to pi interaction (central group as acceptor)
CH/π-2: carbon–hydrogen to pi interaction (central group as donor)
halogen/π: halogen–pi interaction
QM: quantum mechanical
MIF­(QM): molecular interaction field based on quantum mechanical calculations
CSD: Cambridge Structural Database
PDB: Protein Data Bank
SI: Supporting Information
BZ/BZ: benzene–benzene system
CBZ/BZ: chlorobenzene–benzene system
BBZ/BZ: bromobenzene–benzene system
IBZ/BZ: iodobenzene–benzene system
P: parallel orientation
T: T-shaped (perpendicular) orientation
Rcc: distance between centers of aromatic rings
Rxc: distance between halogen atom and aromatic ring center
D: perpendicular distance between aromatic ring planes
θ: angle between normal vectors of aromatic rings
φ: rotational angle around the *z*-axis
BSSE: basis set superposition error
CP: counterpoise correction
CCSD­(T): coupled cluster singles, doubles, and perturbative triples
CBS: complete basis set
MP2: second-order Møller–Plesset perturbation theory
aug-cc-pVDZ: augmented correlation-consistent polarized valence double-ζ basis set
aug-cc-pVTZ: augmented correlation-consistent polarized valence triple-ζ basis set
aug-cc-pVQZ: augmented correlation-consistent polarized valence quadruple-ζ basis set
aug-cc-pVDZ-PP: augmented correlation-consistent polarized valence double-ζ basis set with pseudopotentials
VMD: Visual Molecular Dynamics
Phe: phenylalanine
Tyr: tyrosine
Trp: tryptophan
CONQUEST: Cambridge Structural Database search software
IsoGen: isosurface generation program
IsoStar: intermolecular interaction database and analysis tool
