# Supplementary material for: Three-Dimensional Descriptions of π/π, CH/π, and Halogen/π Interactions Formed between Phenyl Substructures
Source: J Phys Chem B. 2026 Aug 5;130(33):8395–408. doi: 10.1021/acs.jpcb.6c03632 (PMC13502283; doi:10.1021/acs.jpcb.6c03632)
Supplement: Supplementary file 1 [file jp6c03632_si_001.pdf]

Supporting Information

Three-Dimensional Descriptions of π/π, CH/π, and Halogen/π Interactions Formed between Phenyl Substructures

Daichi Hayakawa, Hiroaki Gouda*

Department of Biophysical Chemistry, Showa Medical University Graduate School of Pharmacy
1-5-8, Hatanodai, Shinagawa-ku, Tokyo, 142-8555, Japan

*d-hayakawa@pharm.showa-u.ac.jp

Table of contents

DETAILS OF CSD ANALYSES **S3**

FIGURE S1 **S4**

FIGURE S2 **S5**

FIGURE S3 **S6**

FIGURE S4 **S7**

FIGURE S5 **S8**

FIGURE S6 **S9**

FIGURE S7 **S10**

FIGURE S8 **S11**

DIVISION OF MIF(QM) MAPS (FIGURE S9) **S12**

FIGURE S10 **S13**

FIGURE S11 **S14**

FIGURE S12 **S15**

FIGURE S13 **S16**

FIGURE S14 **S17**

FIGURE S15 **S18**

FIGURE S16 **S19**

FIGURE S17 **S20**

Details of CSD Analyses

In this study, benzene (BZ) molecules containing six hydrogen atoms were defined as the central groups. Chlorobenzene (CBZ), bromobenzene (BBZ), and iodobenzene (IBZ) substructures, with or without hydrogen atoms, were also included as central groups. BZ molecules containing six hydrogen atoms were used as contact groups for BZ central groups, whereas BZ substructures with or without hydrogen atoms were used as contact groups for CBZ, BBZ, and IBZ central groups.

For the analysis of π/π interactions, the centers of the contact search spheres were defined at the centers of the heavy atoms of the central groups (Figure S1a, Table S1). The centers of the contact groups were defined at the centers of the BZ substructures (Figure S1a, Table S1). The search radii were set to 5 and 7 Å for BZ and to 7 Å for CBZ, BBZ, and IBZ.

CH/π interactions in which the CH group of the contact BZ group acts as the donor and the central aromatic group acts as the acceptor were analyzed and are denoted as CH/π-1. The centers of the contact searches were defined at the centers of the aromatic rings of the central groups (Figure S1b, Table S1). The centers of the contact groups were defined at the centers of the CH groups of the BZ substructures (Figure S1b, Table S1). The searching radius was set at 5 Å.

CH/π interactions in which the CH groups of the central aromatic groups act as donors and the contact BZ groups act as acceptors were also analyzed and are denoted as CH/π-2.

The centers of the contact searches were defined at the hydrogen atoms of the CH groups of the central aromatic rings (Figure S1c–e, Table S1). The centers of the contact groups were defined at the centers of the BZ substructures (Figure S1c–e, Table S1). CH groups at the ortho, meta, and para positions of the halobenzene substructures were analyzed separately. The search radius was set to at 5 Å.

For the analysis of halogen/π interactions, the centers of the contact searches were defined at the halogen atoms of the halobenzene substructures (Figure S1f, Table S1). The centers of the contact groups were defined at the centers of the BZ substructures (Figure S1f, Table S1). The search radius was set to at 5 Å.

The detail conditions of CSD searches are listed in Table S2.

Table S1. Details of contact searches.

Type	Range [Å]	Center of center group	Center of contact group
**π/π**	7 or 5	Center of substructure	Center of BZ substructure
**CH/π-1**	5	Center of aromatic ring	Center of CH group of BZ
**CH/π-2**	5	Hydrogen atom of CH group	Center of BZ substructure
**Halogen/π**	5	Halogen atom	Center of BZ substructure

CH/π-1: CH/π interactions where the center groups act as the acceptors.

CH/π-2: CH/π interactions where the center groups act as the donors.

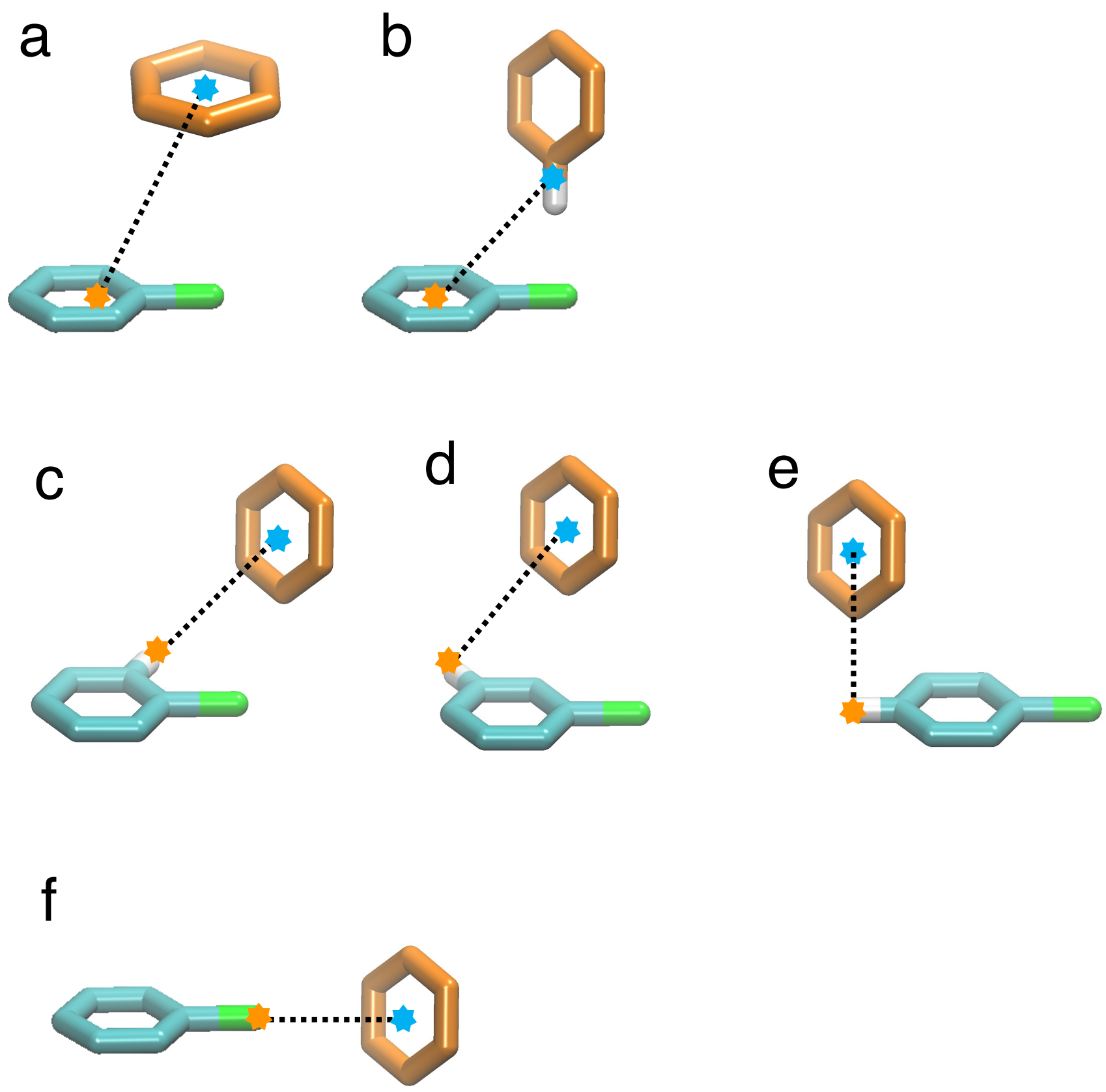

Figure S1. Schematic illustrations of contact search conditions for (a) π/π, (b) CH/π-1, (c) CH/π-2 (ortho), (d) CH/π-2 (meta), (e) CH/π-2 (para), and (f) halogen/π interactions. Orange stars indicate the centers of the search spheres, and blue stars indicate the centers of the contact groups.

Table S2. Detail conditions of CSD searches.

R-factor ≦ 0.05
3D coordinates determined
Only Nondisordered
No errors
Not polymeric
No ions
Only single crystal structures
Only organics

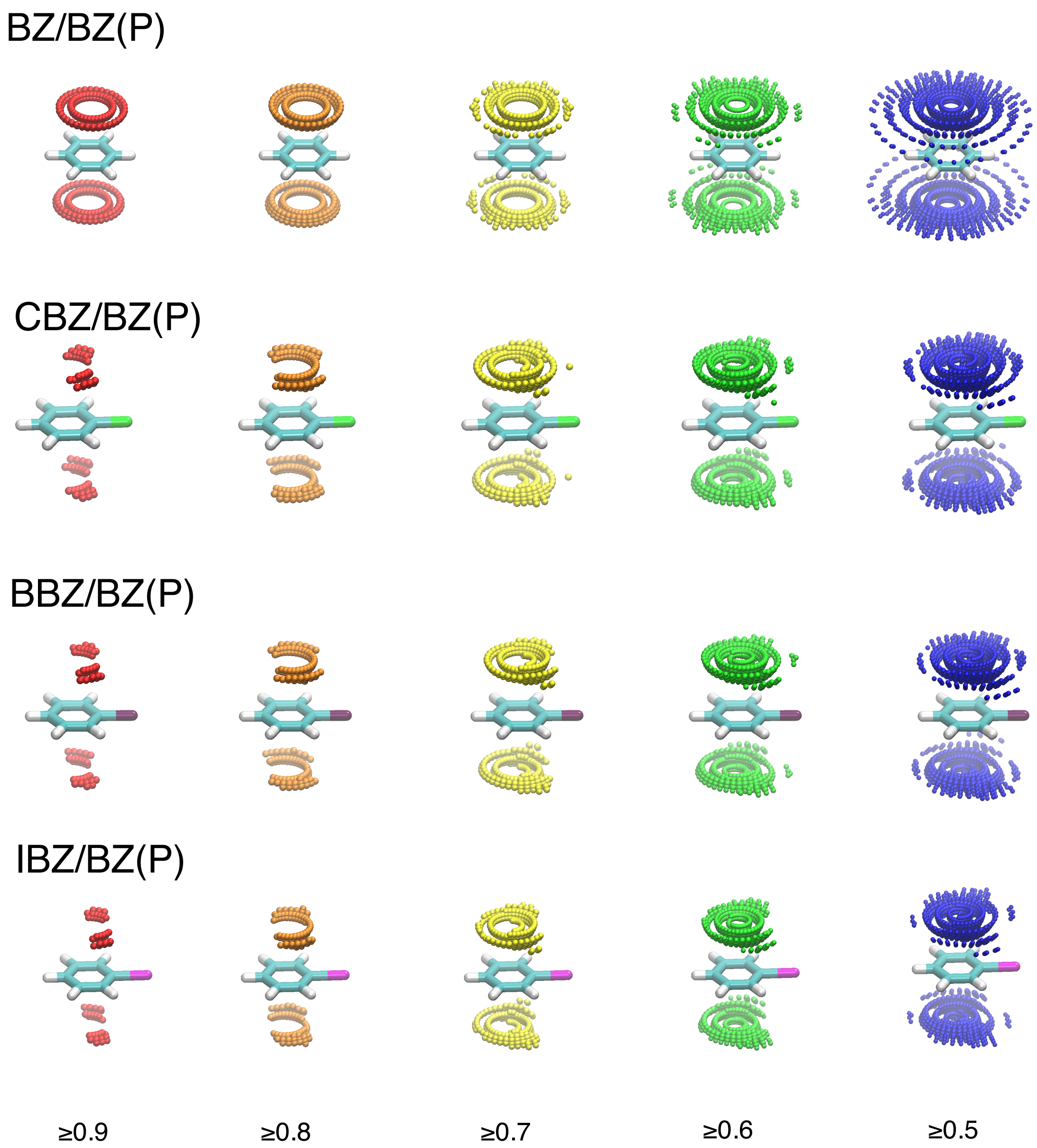

Figure S2. MIF(QM) maps for the BZ/BZ(P), CBZ/BZ(P), BBZ/BZ(P), and IBZ/BZ(P) systems. In the MIF(QM) calculations, the probe BZ molecule was oriented parallel to the target molecule. Regions with normalized energies ≥0.9, 0.8, 0.7, 0.6, and 0.5 are shown as red, orange, yellow, green, and blue spheres, respectively.

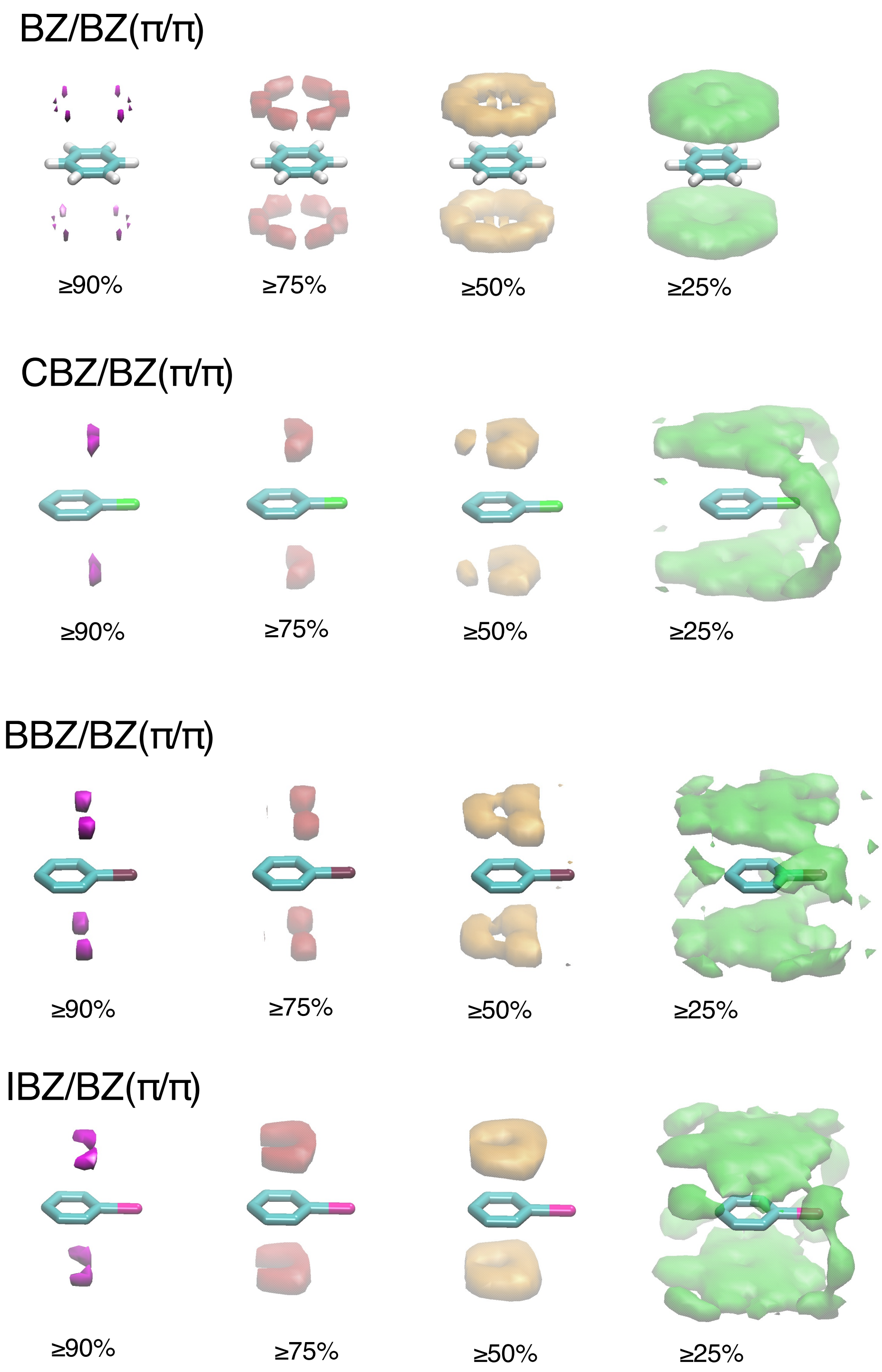

Figure S3. Density(CSD) maps of π/π interactions for BZ, CBZ, BBZ, and IBZ substructures. Density(CSD) values were calculated using contact groups with parallel orientations. Regions with densities ≥90%, ≥75%, 50%, and 25% of the maximum density are shown as magenta, red, orange, and green surfaces, respectively. The search radii were set to 5 Å for BZ and 7 Å for the other systems.

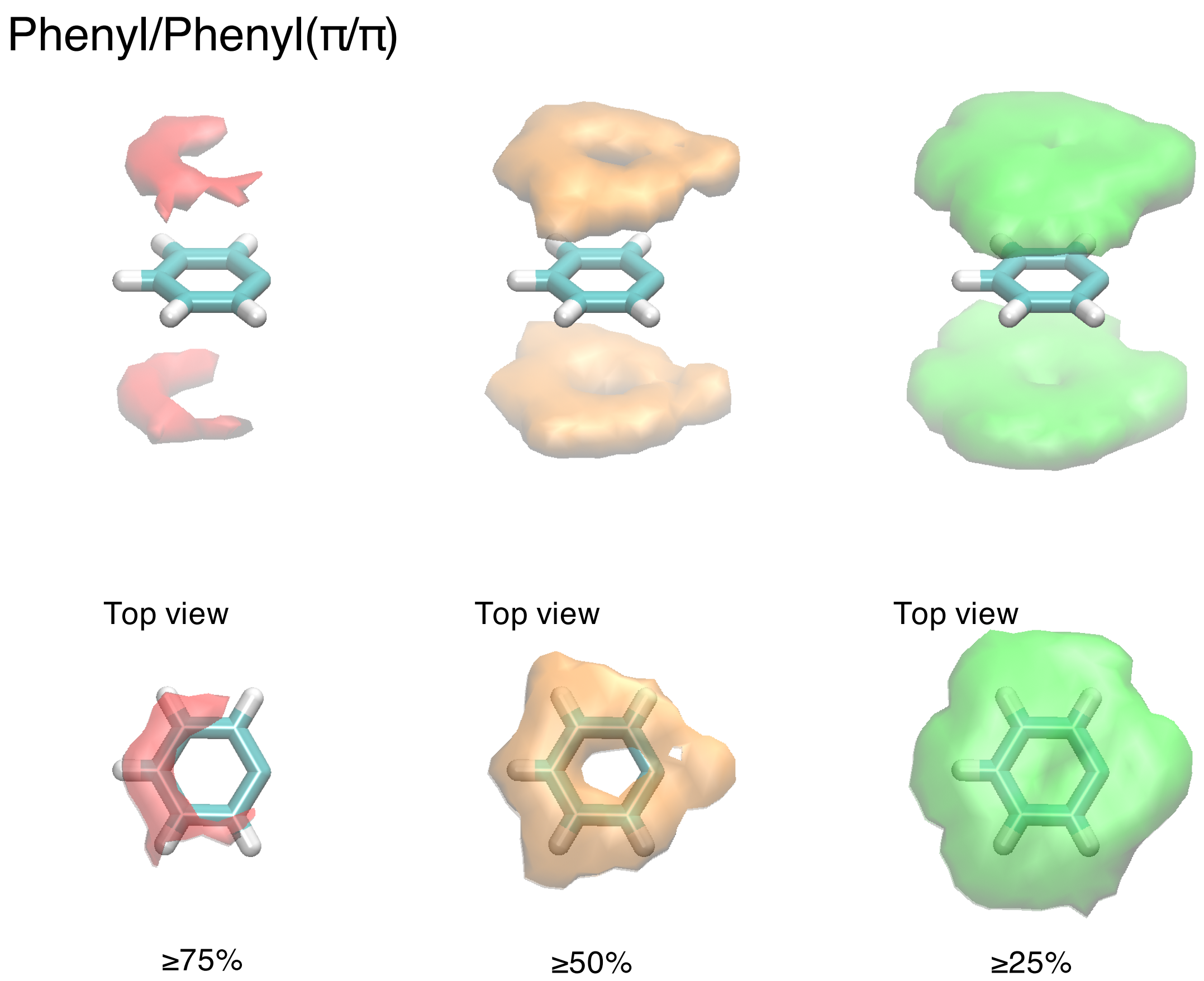

Figure S4. Density(CSD) maps of π/π interactions for phenyl substructures, where the BZ substructure with five hydrogen atoms was defined as both the central and contact group. Density(CSD) values were calculated using contact groups with parallel orientations. Regions with densities ≥75%, 50%, and 25% of the maximum density are shown as red, orange, and green surfaces, respectively. Density(CSD) values were calculated using contact groups within 5 Å of the center of the phenyl substructure.

Phenyl/Phenyl(π/π)

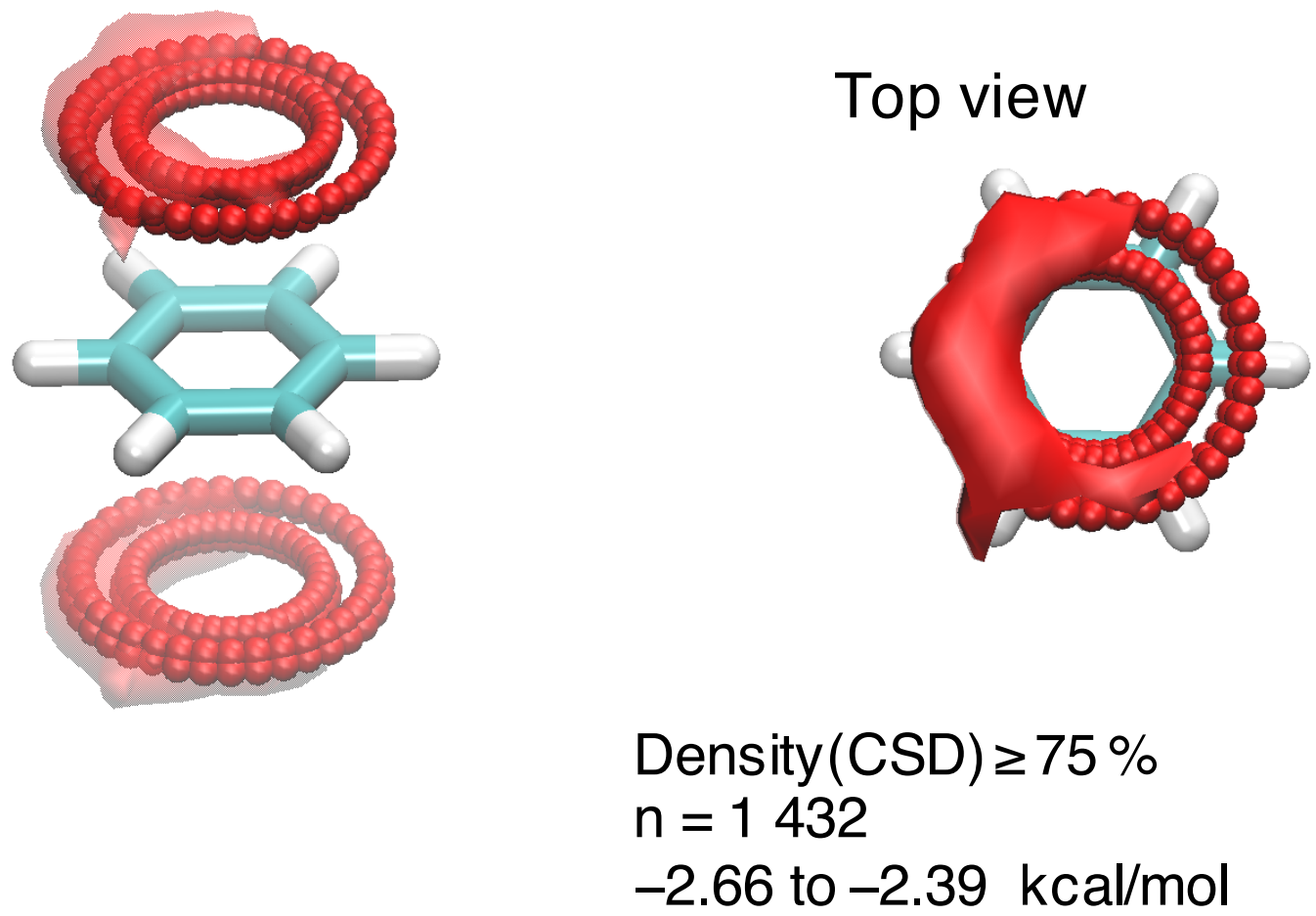

Figure S5. Superimposed MIF(QM) and density(CSD) maps of π/π interactions for phenyl substructures. Grid points with normalized energies ≥0.9 are shown as red spheres, and regions with densities ≥75% .

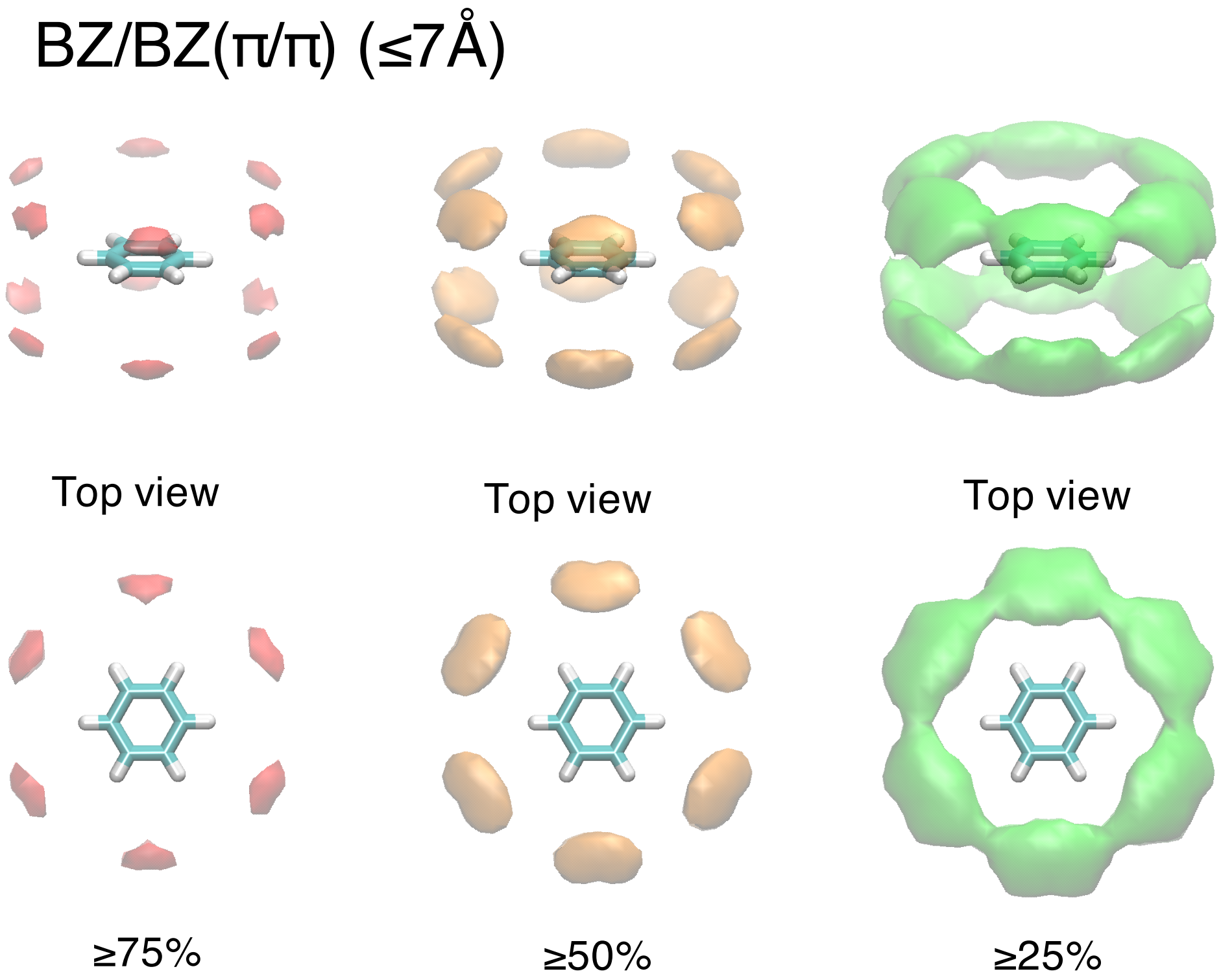

Figure S6. Density(CSD) maps of the π/π interactions for the BZ. The density(CSD) values were calculated using the contact groups with parallel orientations. The search radius was set at 7 Å. Regions with densities ≥75%, 50%, and 25% of the maximum density are shown as red, orange, and green surfaces, respectively. The distance between the center of BZ and the density peak at 75% is 5.6 Å.

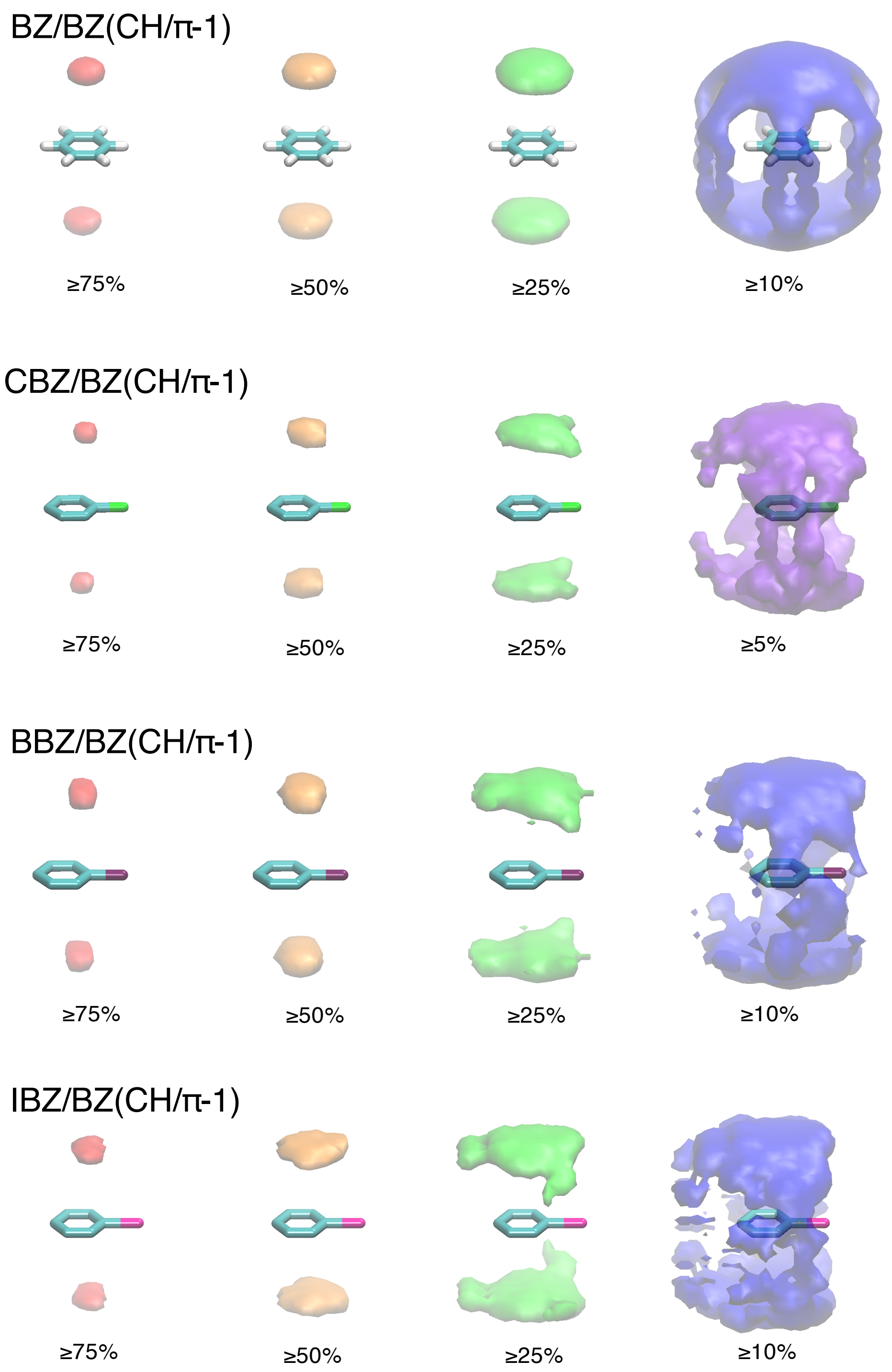

Figure S7. Density(CSD) maps of CH/π-1 interactions for BZ, CBZ, BBZ, and IBZ substructures. Density(CSD) values were calculated using contact groups with perpendicular orientations. The search radius was set to 5 Å. Regions with densities ≥75%, 50%, 25%, 10%, and 5% of the maximum density are shown as red, orange, green, blue, and purple surfaces, respectively.

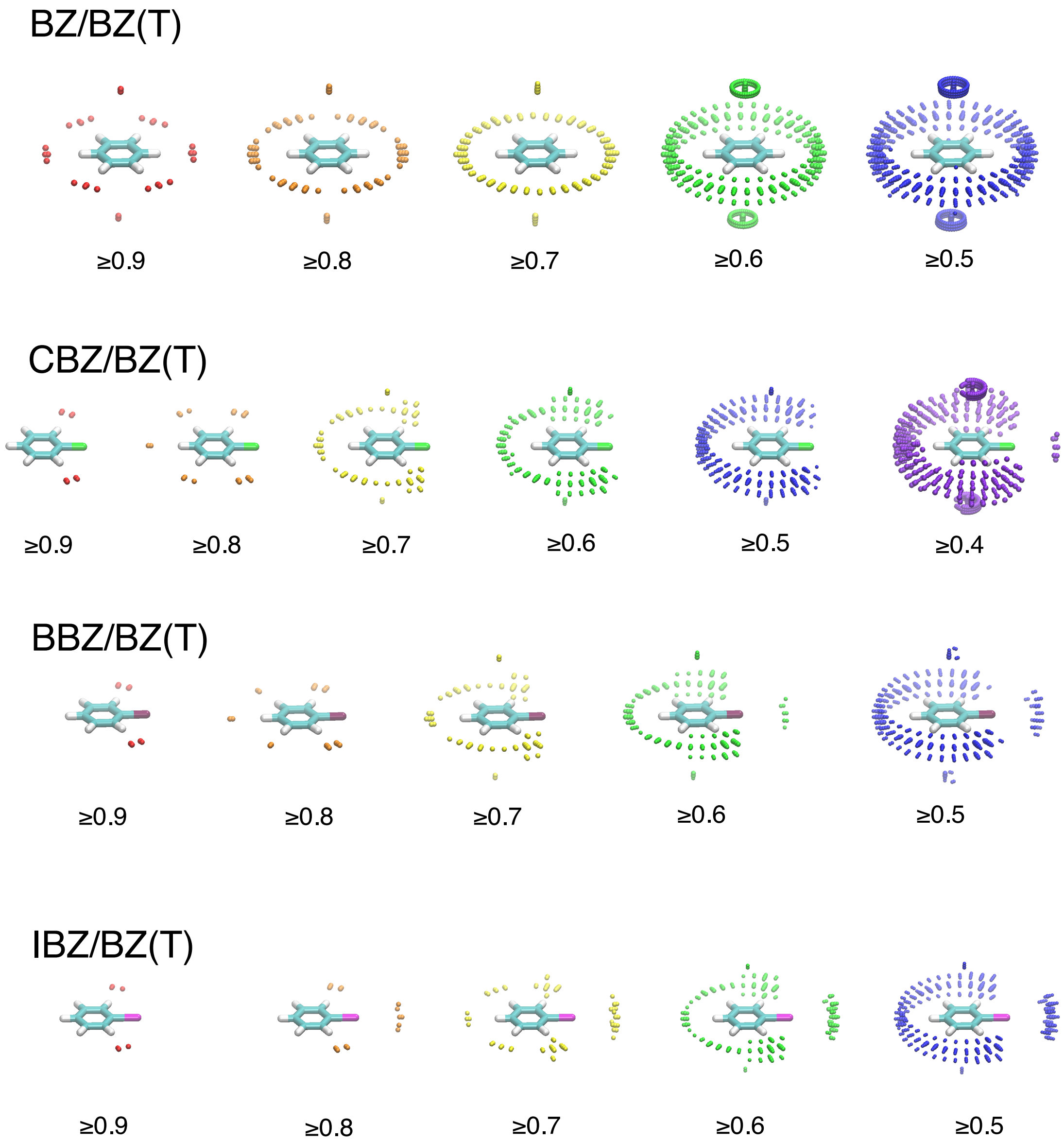

Figure S8. MIF(QM) maps for the BZ/BZ(T), CBZ/BZ(T), BBZ/BZ(T), and IBZ/BZ(T) systems. In the MIF(QM) calculations, the probe BZ molecule was oriented perpendicular to the target molecule. Regions with normalized energies ≥0.9, 0.8, 0.7, 0.6, and 0.5 are shown as red, orange, yellow, green, and blue spheres, respectively.

Division of MIF(QM) Maps.

The full MIF(QM) maps of BZ/BZ(T), CBZ/BZ(T), BBZ/BZ(T), and IBZ/BZ(T) shown in Figure S8 include both CH/π and halogen/π interaction-formable regions. Therefore, these MIF(QM) maps were divided by interaction type using a simple procedure. In this procedure, each grid point was assigned to the nearest heavy atom in the target molecule, excluding the carbon atom bonded to the halogen. The centers of aromatic rings were treated as dummy atoms during this assignment. For example, in the CBZ system, grid points were assigned to five carbon atoms, one chlorine atom, or one dummy atom corresponding to the ring center (Figure S9). Interaction energies were then normalized within each group, yielding seven partitioned MIF(QM) maps for each target molecule. The MIF(QM) maps assigned to the aromatic ring centers were used to analyze CH/π-1 interactions. Similarly, the MIF(QM) maps assigned to carbon atoms and halogen atoms were used to analyze CH/π-2 and halogen/π interactions, respectively.

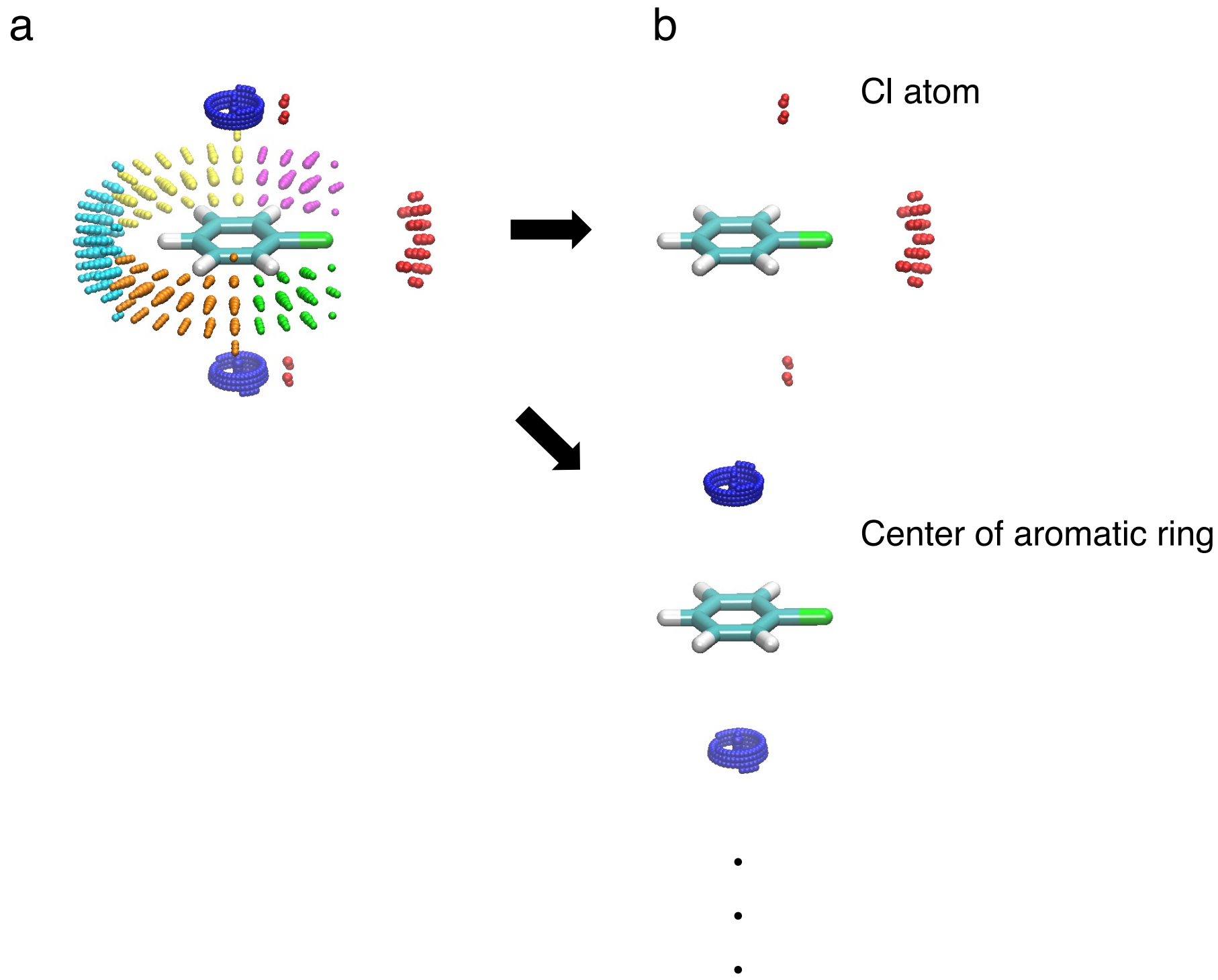

Figure S9. Classification of grid points. (a) Grid points were divided into seven groups.
(b) The resulting MIF(QM) maps were used to analyze CH/π-1, CH/π-2, and halogen/π interactions. In this figure, grid points are colored according to group classification rather than normalized energy.

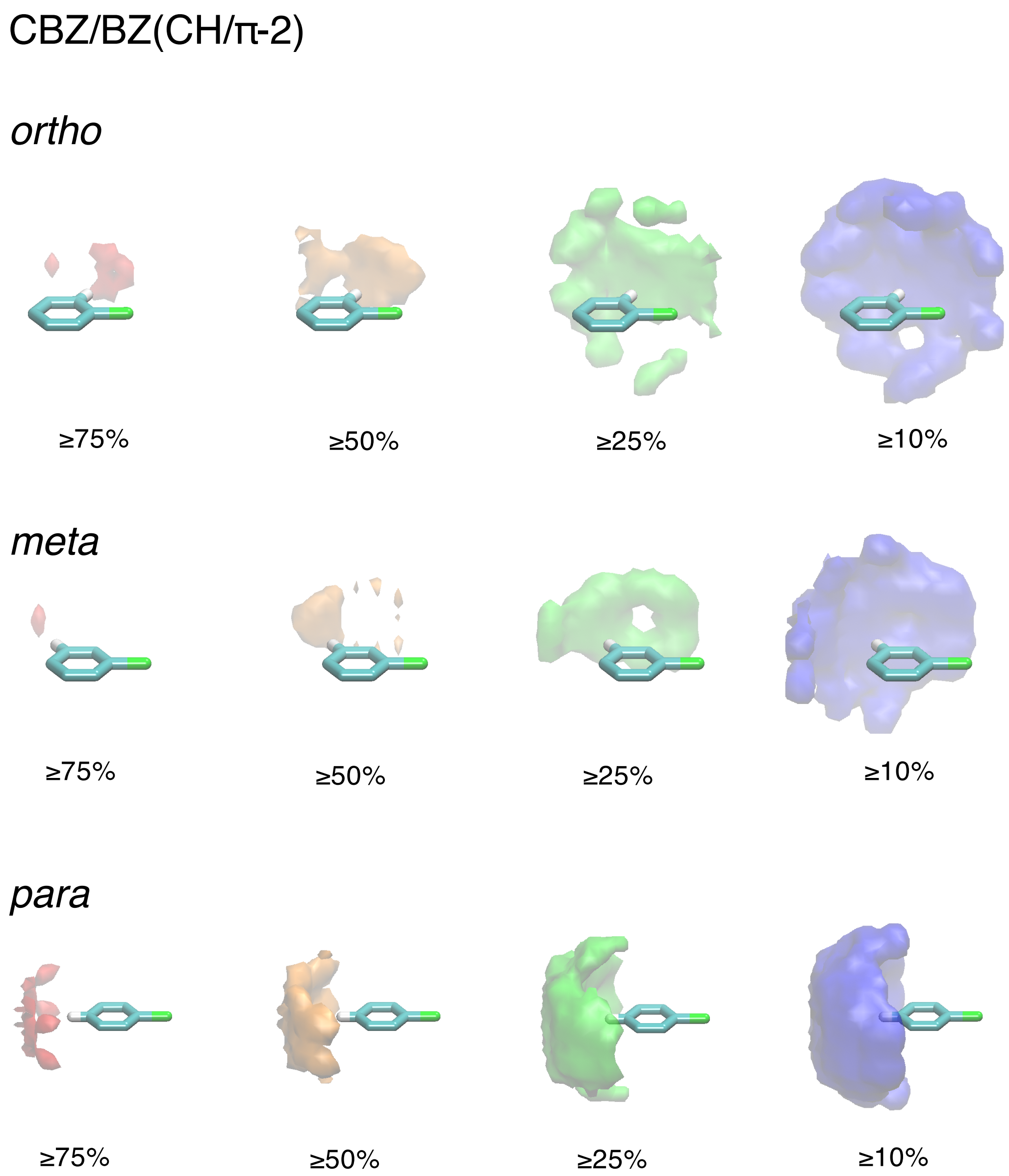

Figure S10. Density(CSD) maps of CH/π-2 interactions around ortho-, meta-, and para-CH groups of CBZ. Density(CSD) values were calculated using contact groups with perpendicular orientations ($\theta \leq 30°$ or $\theta \geq 150°$, $\phi \leq 30°$ or $\phi \geq 150°$). The search radius was set to 5 Å. Regions with densities ≥75%, 50%, 25%, and 10% of the maximum density are shown as red, orange, green, and blue surfaces, respectively.

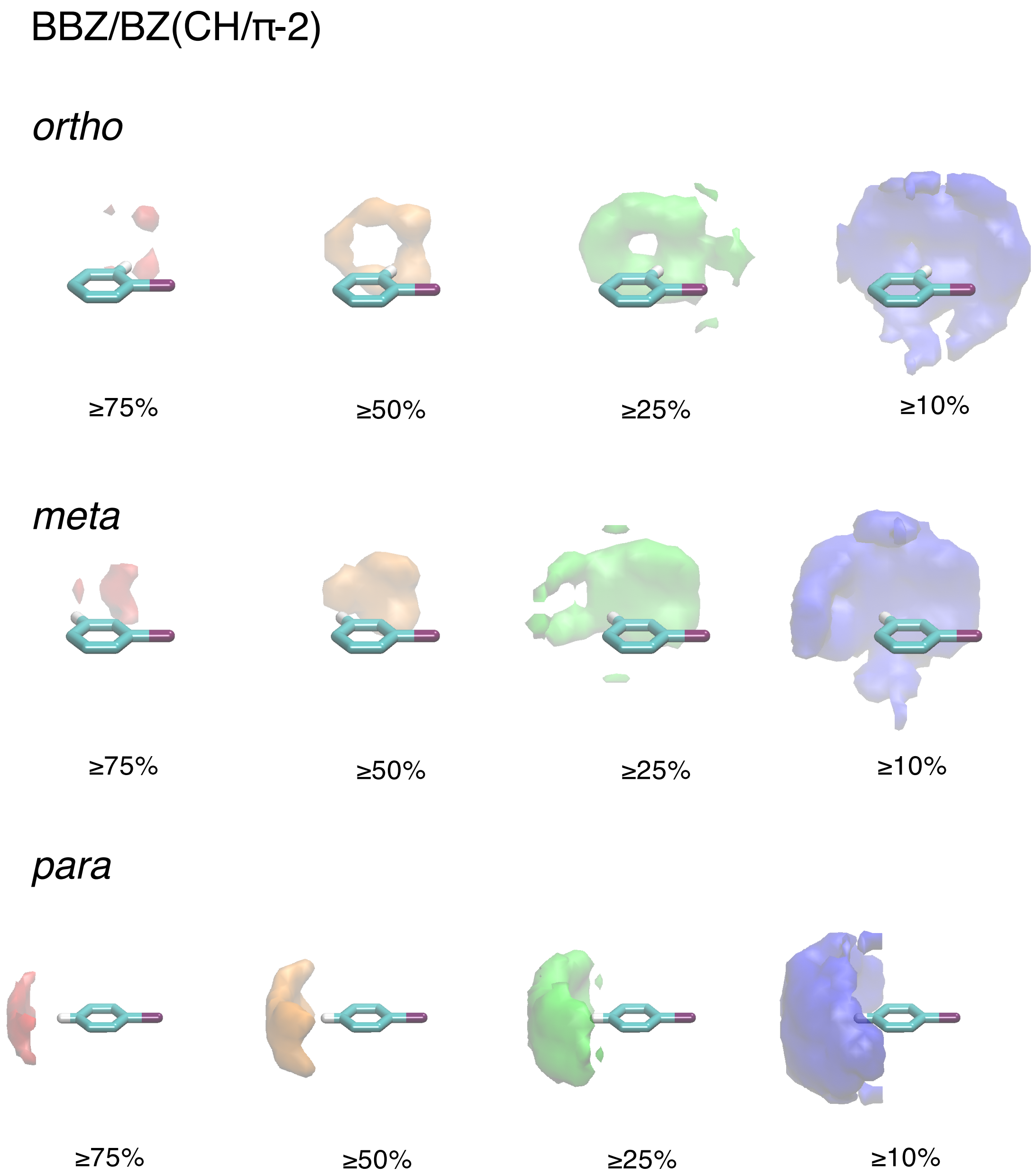

Figure S11. Density(CSD) maps of CH/π-2 interactions around ortho-, meta-, and para-CH groups of BBZ. Density(CSD) values were calculated using contact groups with perpendicular orientations ($\theta \leq 30°$ or $\theta \geq 150°$, $\phi \leq 30°$ or $\phi \geq 150°$). The search radius was set to 5 Å. Regions with densities ≥75%, 50%, 25%, and 10% of the maximum density are shown as red, orange, green, and blue surfaces, respectively.

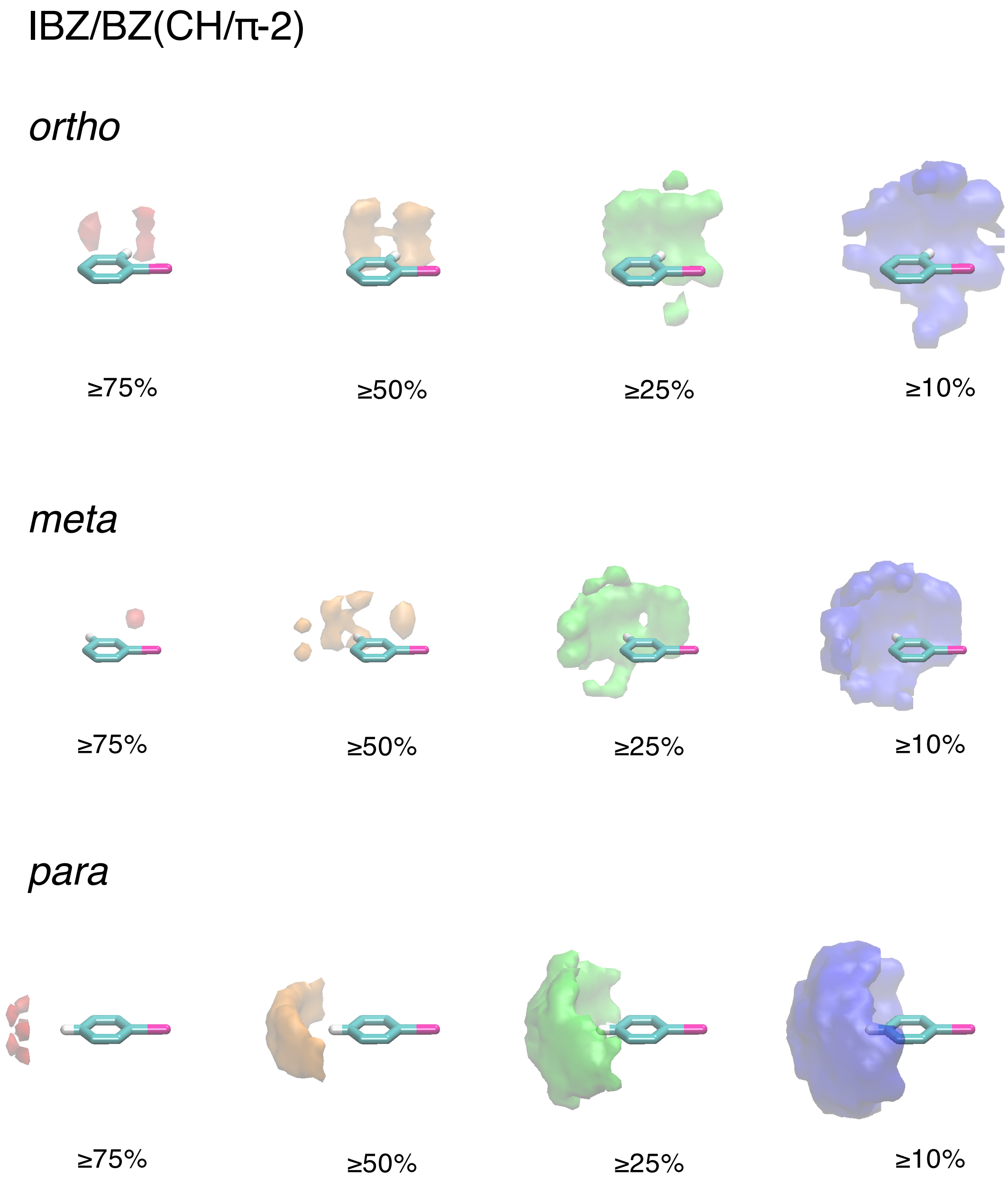

Figure S12. Density(CSD) maps of CH/π-2 interactions around ortho-, meta-, and para-CH groups of IBZ. Density(CSD) values were calculated using contact groups with perpendicular orientations ($\theta \leq 30°$ or $\theta \geq 150°$, $\phi \leq 30°$ or $\phi \geq 150°$). The search radius was set to 5 Å. Regions with densities ≥75%, 50%, 25%, and 10% of the maximum density are shown as red, orange, green, and blue surfaces, respectively.

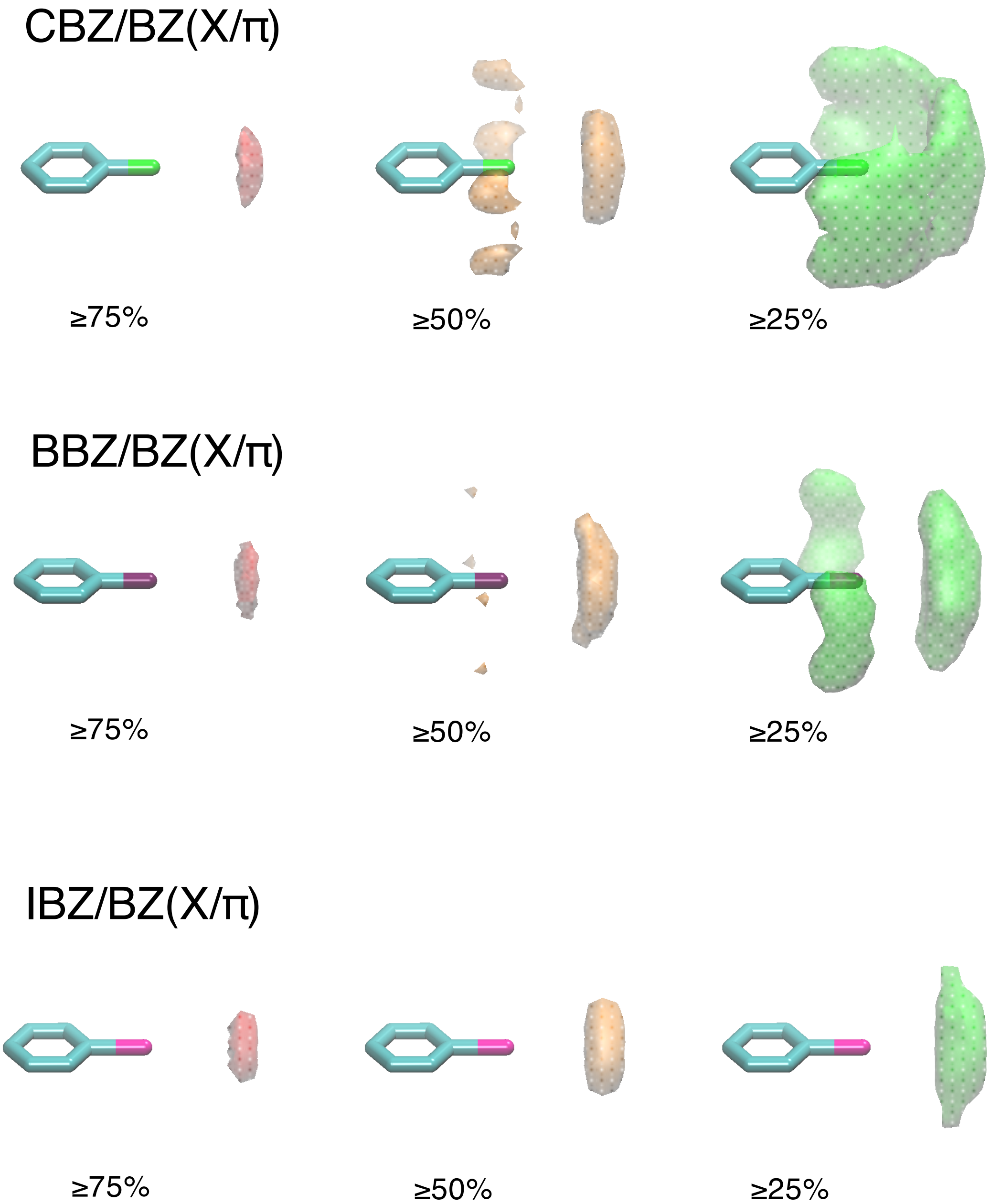

Figure S13. Density(CSD) maps of halogen/π interactions for CBZ, BBZ, and IBZ substructures. Density(CSD) values were calculated using contact groups with perpendicular orientations ($\theta \leq 30°$ or $\theta \geq 150°$, $\phi \leq 30°$ or $\phi \geq 150°$). The search radius was set to 5 Å. Regions with densities ≥75%, 50%, and 25% of the maximum density are shown as red, orange, and green surfaces, respectively.

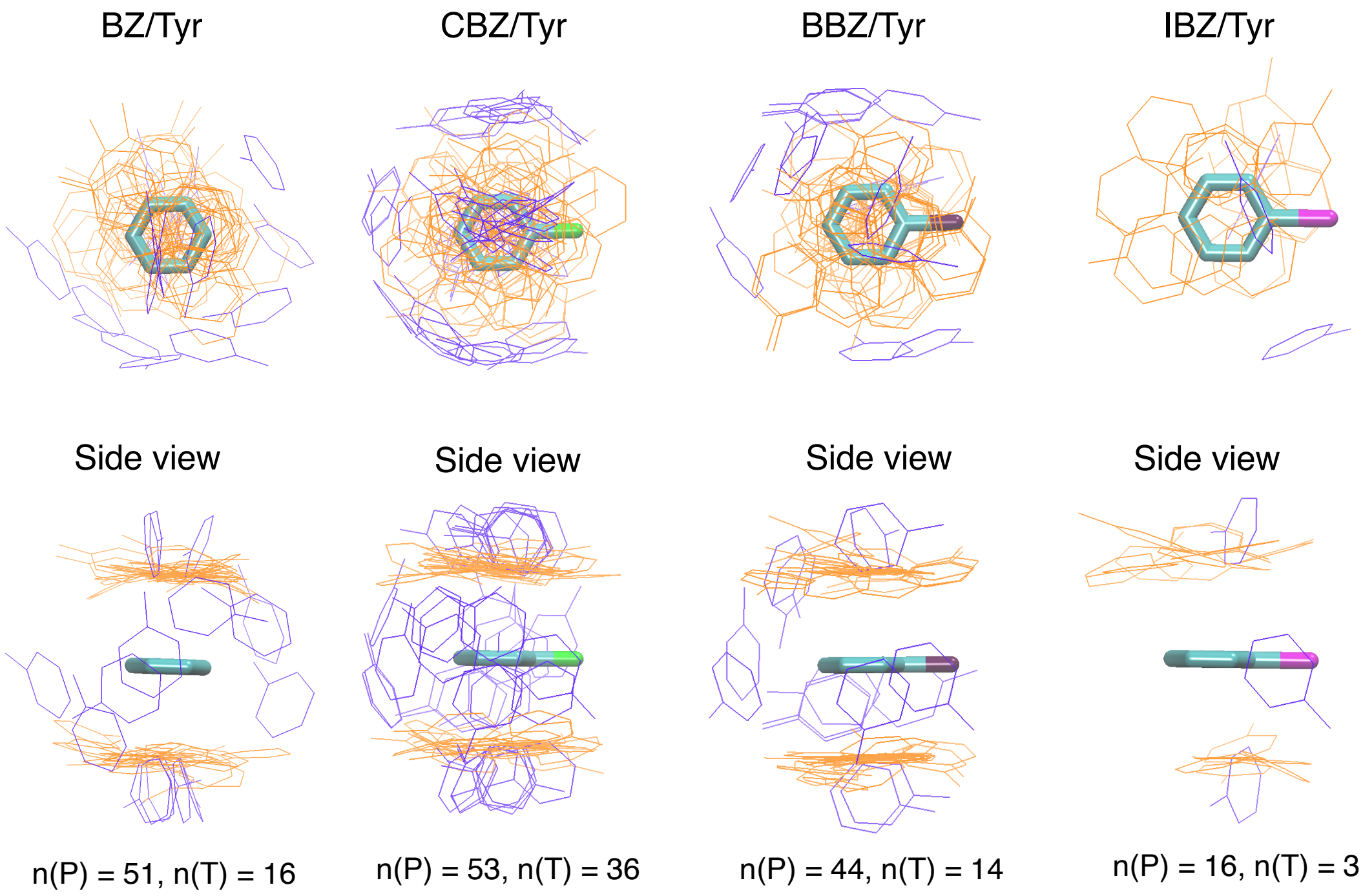

Figure S14. Scatter plots (PDB) of phenol groups from Tyr for BZ, CBZ, BBZ, and IBZ substructures. Contact groups with parallel and T-shaped orientations are shown in orange and purple, respectively. The centers of contact searches were set at the centers of the central groups. n(P) and n(T) denote the numbers of fragments with parallel and T-shaped orientations, respectively.

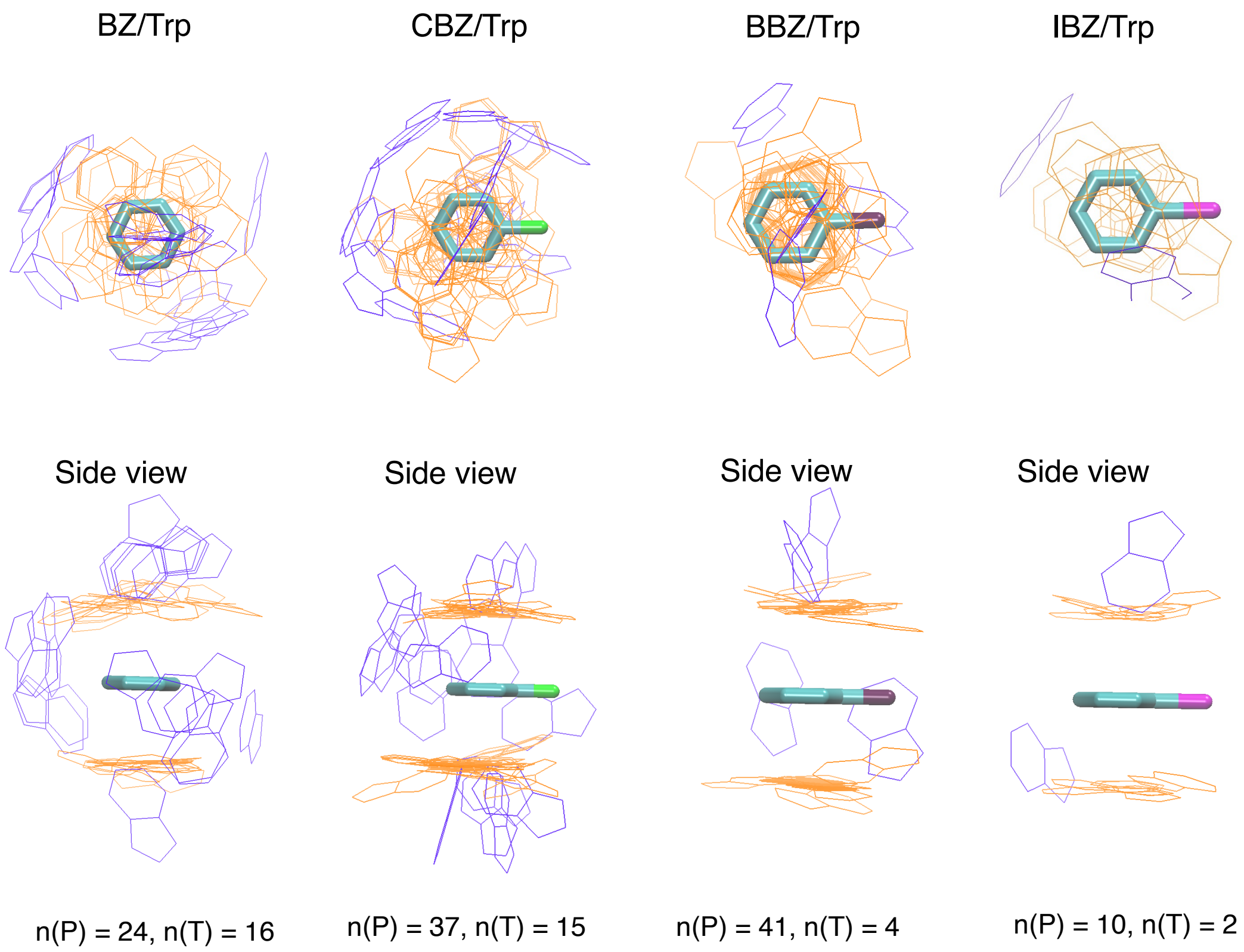

Figure S15. Scatter plots (PDB) of indole groups from Trp for BZ, CBZ, BBZ, and IBZ substructures. Contact groups with parallel and T-shaped orientations are shown in orange and purple, respectively. The centers of contact searches were set at the centers of the central groups. n(P) and n(T) denote the numbers of fragments with parallel and T-shaped orientations, respectively.

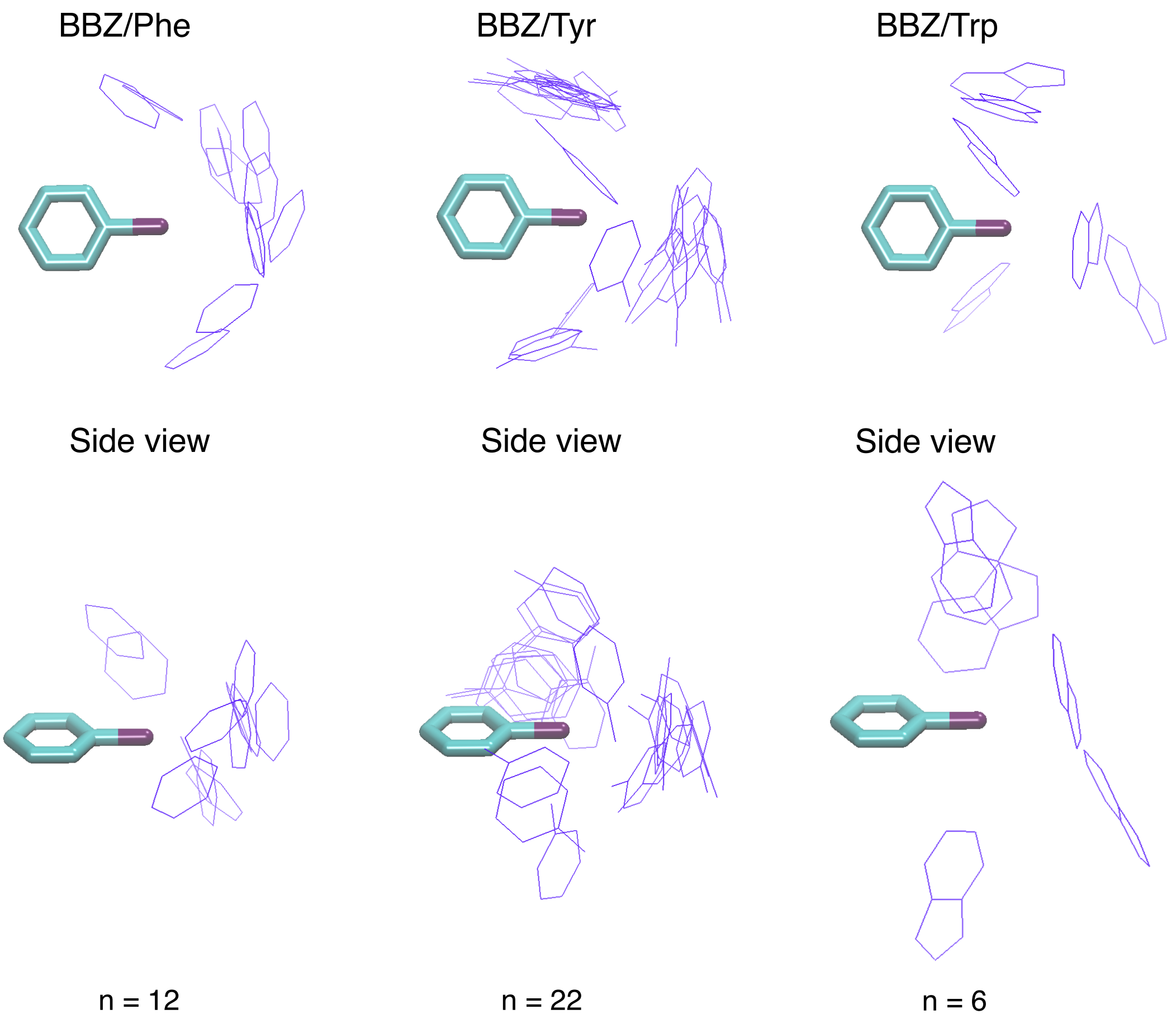

Figure S16. Scatter plots (PDB) of phenyl groups from Phe, phenol groups from Tyr, and indole groups from Trp for BBZ. Contact groups with T-shaped orientations are shown in purple. The centers of contact searches were set at the bromine atoms. n denotes the number of fragments with T-shaped orientations.

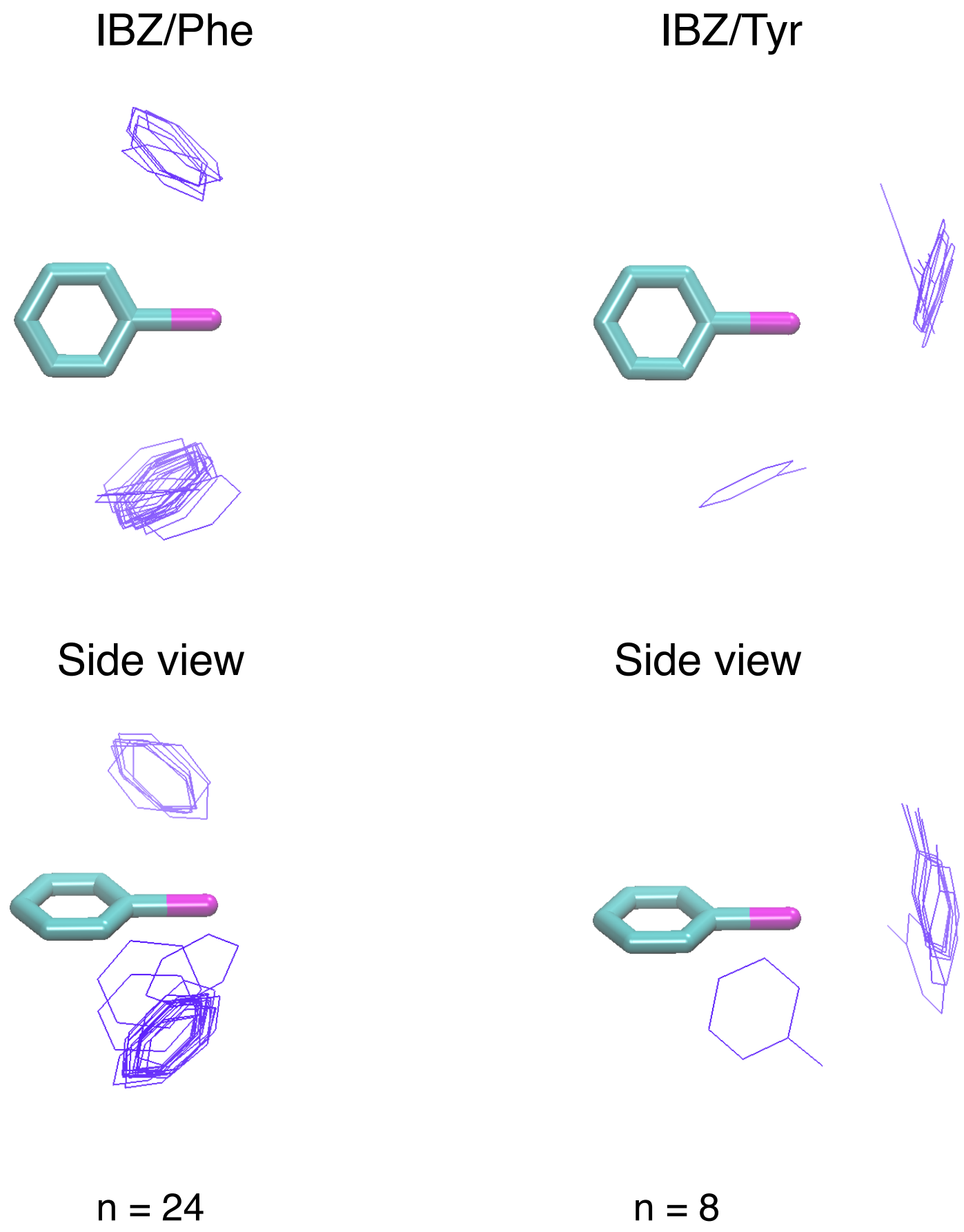

Figure S17. Scatter plots (PDB) of phenyl groups from Phe and phenol groups from Tyr for IBZ. Contact groups with T-shaped orientations are shown in purple. The centers of contact searches were set at the iodine atoms. No indole groups from Trp were observed around IBZ substructures in the dataset. n denotes the number of fragments with T-shaped orientations.
